# Upregulation of *LINC00501* by H3K27 acetylation facilitates gastric cancer metastasis through activating epithelial‐mesenchymal transition and angiogenesis

**DOI:** 10.1002/ctm2.1432

**Published:** 2023-10-23

**Authors:** Rongzhang Dou, Lei Han, Chaogang Yang, Yan Fang, Jinsen Zheng, Chenxi Liang, Jialin Song, Chen Wei, Guoquan Huang, Panyi Zhong, Keshu Liu, Qian Peng, Chunwei Peng, Bin Xiong, Shuyi Wang

**Affiliations:** ^1^ Department of Gastrointestinal Surgery Zhongnan Hospital of Wuhan University Wuhan Hubei China; ^2^ Department of Gastric and Colorectal Surgical Oncology Zhongnan Hospital of Wuhan University Wuhan Hubei China; ^3^ Hubei Key Laboratory of Tumor Biological Behaviors Wuhan Hubei China; ^4^ Hubei Cancer Clinical Study Center Wuhan Hubei China; ^5^ Department of Obstetrics and Gynecology Guangzhou Women and Children's Medical Center Guangzhou Guangdong China; ^6^ Department of Internal Medicine Affiliated Tumor Hospital of Zhengzhou University, Henan Cancer Hospital Zhengzhou China; ^7^ Department of Pathology Zhongnan Hospital of Wuhan University Wuhan Hubei China

**Keywords:** EMT, gastric cancer, LINC00501, metastasis, tumour microenvironment

## Abstract

**Background:**

The molecular mechanism of the significant role of long noncoding RNAs (lncRNAs) in the progression and metastasis of gastric cancer (GC) remains largely elusive. Our objective is to detect overexpressed lncRNA in GC and investigate its role in promoting epithelial‐mesenchymal transition and tumour microenvironment remodel.

**Methods:**

LncRNA differential expression profile in GC was analysed using RNA microarrays. The level of *LINC00501* was evaluated in both GC patient tissues and GC cell lines by quantitative reverse transcription PCR and large‐scale (*n* = 304) tissue microarray. To explore the biological role and regulatory driver of LINC00501 in GC, various experimental techniques including Chromatin isolation by RNA purification (ChIRP), RNA immunoprecipitation (RIP), chromatin immunoprecipitation (ChIP) assay, dual luciferase assays were performed.

**Results:**

Clinically, it was observed that *LINC00501* level was abnormal overexpression in GC tissue and was associated with GC progression and distant metastasis. Gain and loss molecular biological experiments suggested that *LINC00501*, promoted EMT process and angiogenesis of GC. Mechanically, the enrichment of H3K27 acetylation in *LINC00501* promoter region contributed to the increase of *LINC00501* in GC. *LINC00501* transactivated transcription of *SLUG*, by recruiting hnRNPR to its promoter. The growth of GC was inhibited both in vitro and in vivo by suppressing the level of *LINC00501* using pharmacological intervention from the histone acetyltransferase (HAT) inhibitor ‐C646.

**Conclusions:**

This study suggests that *LINC00501* promotes GC progression via hnRNPR/SLUG pathway, which indicates a promising biomarker and target for GC.

## Graphical abstract

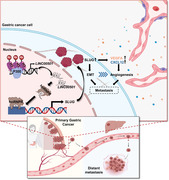

## 1 BACKGROUND

Gastric cancer (GC) is one of the most prevalent cancers and holds the third position among cancer‐related deaths globally.^1^ Despite the improved prognosis of patients with GC, metastasis is still regarded as end‐stage sign of GC and continues to be the primary reason of mortality in GC.^2^, ^3^ By contrast, the molecular mechanism for metastasis in GC is largely unclear.

Metastasis is a complex and successive biological process, known as invasion‐metastasis cascade. During this, tumour cells invade locally through extracellular matrix, intravasate into blood vessels, survive and extravasate into distant organs and colonise in distant organs. During this cascade, epithelial‐mesenchymal transition (EMT), a multistep, complex physiopathological process, is confirmed to play important roles in the both the initial and later stage of metastasis.^4^, ^5^ During the EMT process, there us a decrease in the expression of cell‐cell adhesion protein, mainly the E‐CADHERIN decreases and the level of mesenchymal marker proteins like N‐CADHERIN and VIMENTIN are increased.^6^ The process of EMT is controlled by key transcription factors of EMT (EMT‐TFs) including ZEB1/2, SNAI1, SLUG and TWIST1.^7^ The direct or indirect repression of cell‐cell adhesion by EMT‐TFs and adoption of a migratory and invasive mesenchymal phenotype provides tumour cells the ability to emigrate from primary tumour site to distant organs.^8^, ^9^, ^10^ Moreover, tumour cells in EMT per se can secrete various cytokines to remodel the tumour microenvironment, including promotion of angiogenesis; thus, they collectively accelerate the intravasion of tumour cells and subsequent metastasis.^11^ Therefore, clarifying the underlying mechanism of EMT regulation is important to better understand GC metastasis in a better way.

Long noncoding RNAs (lncRNAs) are a large type of RNA that exceed 200 nucleotides and do not possess any evident ability to encode proteins.^12^ lncRNAs undertakes several functions in EMT and angiogenesis regulation through various mechanisms.^13^, ^14^, ^15^, ^16^, ^17^, ^18^ For instance, *lncRNA‐BX111* upregulates ZEB1 and promotes progression of pancreatic cancer.^19^ *AFAP1‐AS1* decreases ubiquitination and degradation of c‐Myc protein and thus accelerates progression of lung cancer.^20^ *RAB11B‐AS1* enhances the angiogenesis process and metastasis in breast cancer by enhancing translation of *VEGFA* and *ANGPTL4*.^21^ Nevertheless, the regulatory roles of the aberrantly expressed lncRNAs in EMT and angiogenesis in GC remain largely unclear.

Herein, we discovered a lncRNA called *LINC00501*, which is overexpressed in gastric tumour tissues compared with the adjacent nontumour tissues by performing genome‐wide lncRNAs profile microarray screening, qRT‐PCR, and large‐scale tissue microarray assay. Further analysis revealed that *LINC00501* was elevated in advanced GC patient tissues and correlated with metastasis status and poorer outcomes. Furthermore, the assessment of *LINC00501* function assays indicated *LINC00501* facilitated EMT, invasion, migration and angiogenesis in GC. Mechanically, upstream analysis of *LINC00501* revealed that *LINC00501* was upregulated in GC because of the enrichment of H3K27ac mediated by P300 in its promoter. *LINC00501*, which is situated in the nucleus, increases the expression of *SLUG* by enlisting the hnRNPR protein to the promoter area of *SLUG* thereby stimulating EMT and angiogenesis. Importantly, inhibition of *LINC00501* by lentivirus or P300 inhibitor C646 significantly suppressed tumour growth of GC, which indicates that targeting P300/*LINC00501* axis may be an optional target for GC.

## 2 METHODS AND MATERIALS

### 2.1 Patients and clinical samples

Six pairs of fresh gastric cancer tissues was collected for microarray analysis, 40 sets of fresh primary GC tissue samples (cohort 1), and a large‐scale GC tissue microarray sample (cohort 2, *n* = 304) from patients who had surgery at our clinical centre, the diagnosis of gastric cancer of all samples was confirmed by two pathologists. All included patients were devoid from chemotherapy or radio‐therapy before surgery. The written informed consent of patients was obtained before surgery operation. After obtaining approval from the Medical Ethical Committee of our centre (Zhongnan Hospital, Wuhan, Hubei, PR China), this study was conducted.

### 2.2 Microarray analysis

Microarray analysis was conducted using with Arraystar Human LncRNA Microarray V4.0 on six sets of gastric cancer samples and matched adjacent normal tissues. Following the normalisation of the original data, the significant lncRNAs were determined. The array data were available via GSE193109.

### 2.3 Polysomes fraction

Polysomes fraction was modified and performed as described.^22^ Briefly, circa 2 × 10^7^ cells were cocultured with RPMI 1640 medium containing 100 μg/mL cycloheximide (CHX) for 20 min at 37°C. Then the cell was collected with centrifugation and lysed with specific lysis buffer consists of 15 mM MgCl2, 0.3 M NaCl, 1% Triton X‐100, 1 mg/mL heparin, 100 μg/mL cycloheximide, 1× proteinase inhibitor and RNase inhibitor, 15 mM Tris‐HCl. After 10 min, the mixture was centrifuged for 10 min at the speed of 13 000 rpm. The supernatant contained equal amount of RNA (100–300 μg) was retracted and added into 10−50% continuous, linear sucrose gradients. After centrifugation with a speed of 35 000 rpm for 190 min, the RNA distribution was monitored with an ISCO fractionator (Brandel, Inc.).

**HEADLINES**

Distant metastasis is still the leading cause of gastric cancer, which is dependent on tumour cell invasive phenotype and tumour microenvironment.

Epithelial‐mesenchymal transition (EMT) is a pathological process in tumour cells, which not only enhances tumour invasive ability but also remodels tumour microenvironment.

LINC00501 regulates the EMT process and thus promotes tumour cell invasion and angiogenesis, ultimately enhancing gastric cancer progression.

### 2.4 RNA pulldown/mass spectrometry

RNA pulldown assay kit was purchased from the BersinBio CO., Ltd (BersinBio, Guangzhou, China) followed the instruction. Biotin‐labelled *LINC00501* and LacZ probe were synthesised by BersinBio and followed by incubation with extracts from gastric cancer cells. Next, the proteins extract from the pulldown were separated using an SDS‐PAGE gel and subsequently silver‐stained for band visualisation. Finally, the specific band was cut out followed by mass spectrometry or western blot.

### 2.5 Subcellular fractionation analyses

Around five million cells were digested, followed by a PBS wash and centrifugation. The cells that were gathered were broken down using 800 μL hypotonic lysis buffer (Cayman Chemical, USA) and 10% Nonidet P‐40 (Cayman Chemical, USA) was added in 0.4% concentration; after being centrifuged, 450 μL of the supernatant was taken and marked as a cytoplasmic fraction, and the remaining deposit was washed for three times and retained as the nucleus fraction. In the further qRT‐PCR analysis, for the cytoplasmic fraction, *β‐ACTIN* was selected as control; for the nucleus fraction, U6 was selected as control.

### 2.6 Primer and siRNA/shRNA sequence

The sequence of qRT‐PCR primers and siRNA/shRNA can be retrieved from Supplementary Table S1 and Supplementary Table S2.

## 3 RESULTS

### 3.1 *LINC00501* correlates with advanced GC stage and metastasis

**FIGURE 1:**
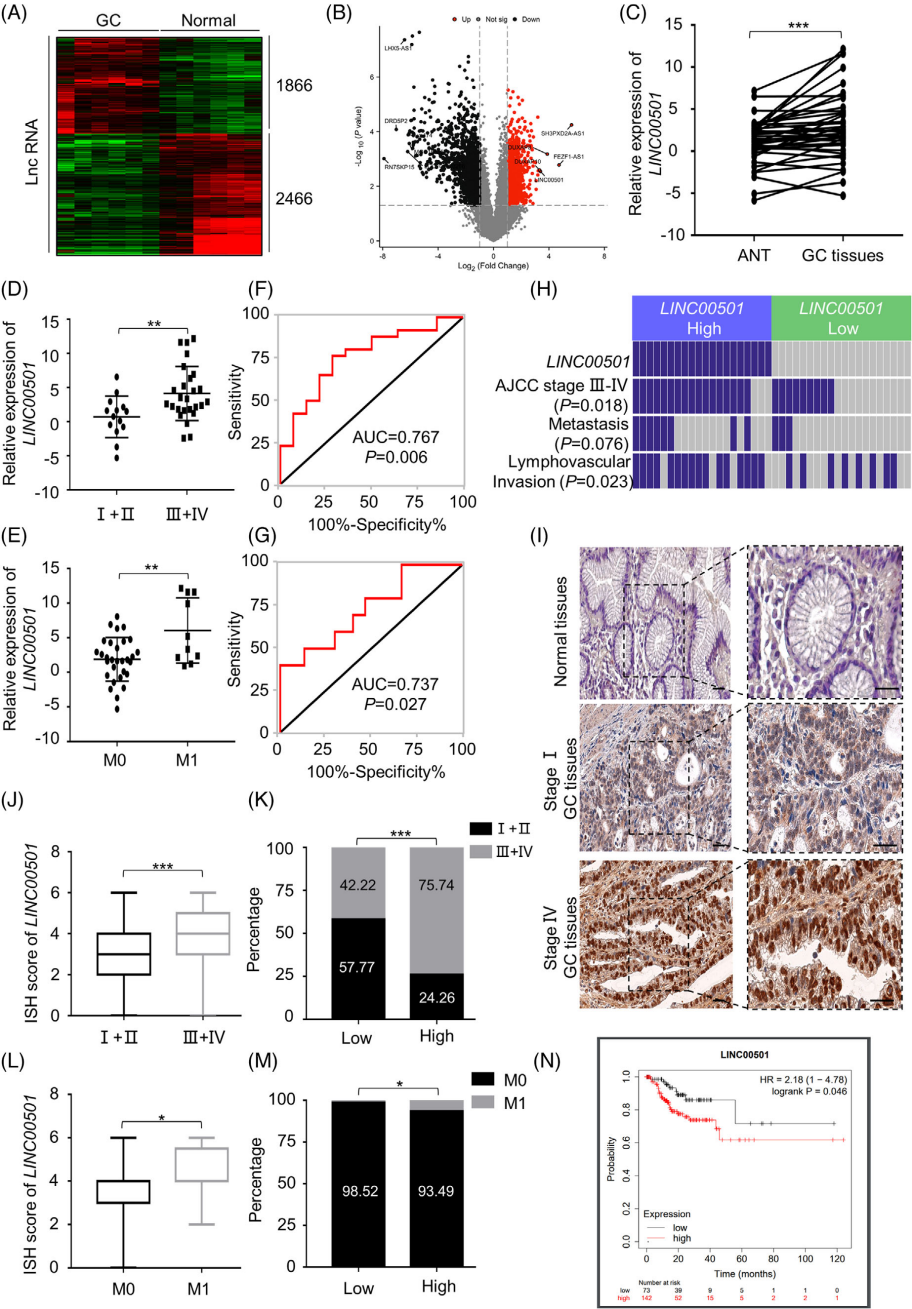
*LINC00501* correlates with advanced GC stage and metastasis. (A, B) The differentiated expression of lncRNAs in 6 pairs of GC tissues and adjacent normal stomach tissues was analysed using microarray analysis. (C) qRT‐PCR analysis of *LINC00501* expression level in 40 pairs of fresh GC tissues and matched normal adjacent tissues. (D) qRT‐PCR analysis of *LINC00501* expression level in patients with GC of stages I, II, III or IV. (E) qRT‐PCR analysis of *LINC00501* expression level in patients with nonmetastatic (M0) and metastatic (M1) GC. (F) Receiver operating characteristic (ROC) analysis was conducted to assess the ability of *LINC00501* to distinguish stages III and IV from stages I and II in GC. (G) ROC analysis was conducted to assess the ability of *LINC00501* to distinguish metastasis (M1) from nonmetastasis (M0). (H) Chi‐square analysis of AJCC stage, positive or negative lymphovascular invasion, and synchronous metastasis status between high and low expression of *LINC00501* in GC in cohort 1 (*n* = 40). (I) Representative in situ hybridisation (ISH) staining for *LINC00501* expression in GC with various stages (I, IV) and adjacent gastric tissues in tissue microarrays (cohort 2; *n* = 304). Scale bar, 100 μm. (J) Analysis of ISH staining score of *LINC00501* in stages I and II and stages III, IV patients with GC. (K) Percentage of stages I and II and stages III and IV patients with GC with high and low expression of *LINC00501* in cohort 2 (*n* = 304). (L) Analysis of ISH staining score of *LINC00501* in patients with GC with nonmetastasis (M0) and metastasis (M1). (M) Percentage of patients with GC with nonmetastasis (M0) and metastasis (M1) with high and low expression of *LINC00501* in cohort 2 (*n* = 304). (N) Relapse‐free survival was analysed and compared between patients with high and low levels of *LINC00501* based on online website Kaplan‐Meier plotter(http://kmplot.com/analysis/index.php?p = service&cancer = pancancer_rnaseq). The hazard ratio and 95% CI were calculated. *p < .05, **p < .01, ***p < .001.

In order to comprehend the regulatory profile of lncRNAs in GC, our team performed a microarray analysis on six sets of GC patient tissues and corresponding normal tissues (Figure 1A and B, Supplementary Table S3, uploaded to GEO dataset, accession: GSE193109). Overall, 1866 upregulated and 2466 downregulated lncRNAs were identified. LINC00501, one of the top 5 dysregulated lncRNAs, displayed a significant 10.91‐fold elevation in its expression level when compared to the surrounding noncancerous tissues, considering *LINC00501* has been reported to show the potential to become gastric cancer biomarker,^23^ this result sparked our interest for further study (Supplementary Figure S1A). The RNA characteristics of *LINC00501* were also analysed and indicated *LINC00501* exhibited low‐coding potential and high expression in GI cancers (Supplementary Figure S2A–C). Additionally, a total of 40 sets of GC tissue (cohort 1) were gathered and subjected to qRT‐PCR analysis. The findings validated that *LINC00501* exhibited increased expression in stomach cancer tissues (Figure 1C). Additional examination indicated that in addition to the increased expression of LINC00501 in GC, it was notably elevated in stage III–IV GC tissues (*p* < .01). Moreover, *LINC00501* level in GC tissues was markedly higher in patients with metastasis (Figure 1D and E, Supplementary Table S4). The chi‐square analysis of clinicopathological parameters indicated that *LINC00501* level was positively correlated with advanced stage and lymphovascular invasion (Figure 1H). Based on this observation, we conducted an analysis of receiver operating characteristic (ROC) analysis to assess the capability of *LINC00501* in distinguishing advanced stage GC from other stages (Figure 1F). With a cutoff of 1.728, the AUC was 0.767. The accuracy rates for sensitivity and specificity were 76.92% and 71.43% correspondingly. To assess the predictive value of *LINC00501* in M0/M1 GC tissues, ROC curve analysis was conducted as described above. The AUC was 0.737 (Figure 1G). To further validate these observations, in situ hybridisation (ISH) was conducted on extensive 304 paraffin‐embedded GC samples (cohort 2). Consistent with cohort 1, GC tissues exhibited high expression of *LINC00501* (Figure 1I), and the high levels of *LINC00501* were positively correlated with advanced stage and metastasis status of GC (Figure 1J–M, Supplementary Table S5). In cohort 2, Supplementary Figures showed that *LINC00501* had a consistent predictive value for the advanced stage and metastasis status of GC (Supplementary Figure S3A–C). More importantly,^24^ high *LINC00501* level was positively correlated with poorer recurrence‐free survival (RFS; hazard ratio 2.18, 95% CI: 1.00–4.78; Figure 1N, Supplementary Figure S3D). Collectively, these results suggested that *LINC00501* is highly elevated in GC tissues, and the high levels of *LINC00501* are associated with progression and metastasis of GC.

### 3.2 *LINC00501* is an oncogene and promotes EMT

**FIGURE 2:**
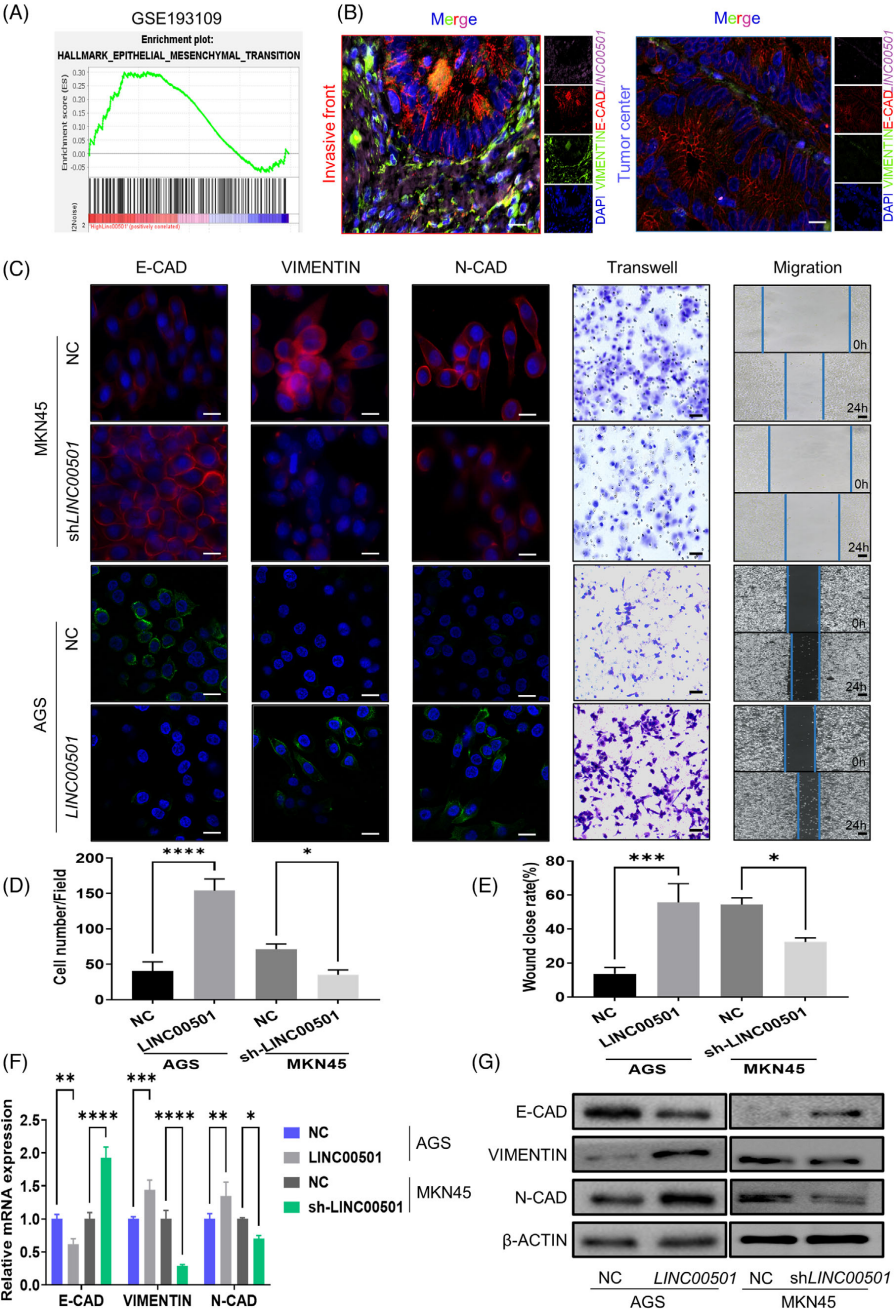
*LINC00501* is an oncogene and promotes EMT. (A) GSEA analyses were conducted to assess the correlation between *LINC00501* and genes related to the EMT process in patients with high and low *LINC00501* expression (GSE193109). (B) Representative FISH/IF staining for the distribution of *LINC00501*, E‐CADHERIN and VIMENTIN in invasive front of GC tissues. Scale bar, 20 μm. (C) The transformation of EMT characteristics in response to the gain and loss of *LINC00501* in AGS and MKN45 cell lines. Immunofluorescence, scale bar, 20 μm. Transwell scale bar, 50 μm. Migration scale bar, 150 μm. (D) The number of migrated cells in transwell invasion assay was calculated in five random fields in AGS‐NC/*LINC00501* and MKN45‐NC/sh‐*LINC00501* cell lines. (E) Wound closure rate in AGS‐NC/*LINC00501* and MKN45‐NC/sh‐*LINC00501* cell lines was calculated to investigate the effect of *LINC00501* on the migratory ability of GC cells. (F) qRT‐PCR analysis of E‐CADHERIN, N‐CADHERIN and VIMENTIN in AGS‐NC/*LINC00501* and MKN45‐NC/sh‐*LINC00501* cell lines. (G) Western blot analysis of E‐CADHERIN, N‐CADHERIN and VIMENTIN in AGS‐NC/*LINC00501* and MKN45‐NC/sh‐*LINC00501* cell lines.

Considering the important role of EMT in GC metastasis, GSEA was conducted. This revealed that EMT hallmarks were highly enriched in patients with high *LINC00501* expression levels (Figure 2A). Moreover, a FISH assay was conducted on GC tissues. It revealed that *LINC00501* was correlated with EMT markers (Figure 2B). To further elucidate the role and mechanism of *LINC00501* in EMT, a series of loss and gain experiments were conducted in GC cell lines. Considering the lowest and highest expression of *LINC00501*, respectively, AGS and MKN45 cell lines were selected for further experiments (Supplementary Figure S4A). The stable *LINC00501*‐overexpressed AGS (AGS‐*LINC00501*) and *LINC00501‐*knockdown MKN‐45 (MKN45‐sh‐*LINC00501*) cell lines were constructed using lentivirus. The efficiency of overexpression and knockdown was verified using qRT‐PCR (Supplementary Figure S4B and C). Transwell cell invasion and wound‐healing experiments demonstrated that compared with negative control (NC), *LINC00501* per se significantly enhanced the migration and invasion ability of GC cells, whereas the loss of *LINC00501* had an opposite effect (Figure 2C–E). Meanwhile, qRT‐PCR and western blot analyses indicated upregulation of N‐CADHERIN and VIMENTIN and downregulation of E‐CADHERIN when *LINC00501* was overexpressed. However, *LINC00501* knockdown inhibited the EMT process (Figure 2F and G). In summary, *LINC00501* promoted migration, invasion and EMT process in GC cells.

### 3.3 SLUG is required for *LINC00501*‐induced EMT

**FIGURE 3:**
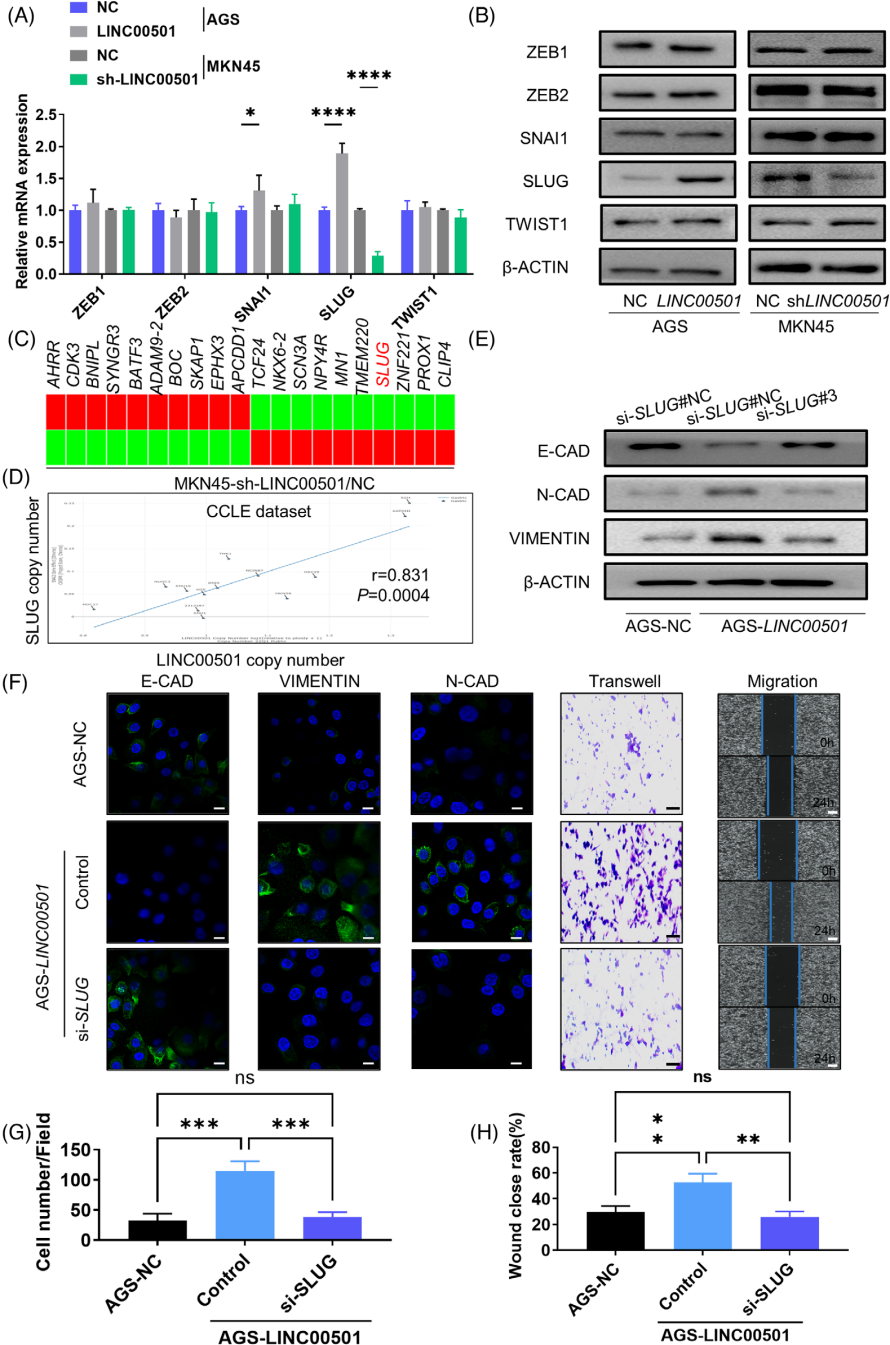
SLUG is required for *LINC00501*‐induced EMT. (A) qRT‐PCR analysis of EMT‐TFs in AGS‐NC/*LINC00501* and MKN45‐NC/sh‐*LINC00501* cell lines. (B) Western blot analysis of E‐CADHERIN, N‐CADHERIN and VIMENTIN in AGS‐NC/*LINC00501* and MKN45‐NC/sh‐*LINC00501* cell lines. (C) Heatmap revealed the change in the expression of representative gene in MKN45 cells after *LINC00501* knockdown. *SLUG* is indicated with red font. (D) The correlation between the expression of *LINC00501* and *SLUG* was analysed using Cancer Cell Line Encyclopedia (https://sites.broadinstitute.org/ccle/). (E) Western blot analysis of E‐CADHERIN, N‐CADHERIN and VIMENTIN in AGS‐*LINC00501* cells with or without *SLUG* knockdown. (F) The transformation of EMT characteristics in response to the knockdown of *SLUG* in AGS‐*LINC00501* cells. Immunofluorescence, scale bar, 20 μm. Transwell scale bar, 50 μm. Migration scale bar, 150 μm. (G) The number of migrated cells through the transwell was calculated in AGS‐*LINC00501* cells cotransfected with *SLUG*‐siRNA or NC. (H) Quantification of wound closure rate in AGS‐*LINC00501* cells cotransfected with *SLUG*‐siRNA or NC.

To assess the regulation of *LINC00501*‐induced EMT process, qRT‐PCR and western blot were performed to investigate the alteration in the expression of transcriptional factors related to EMT (EMT‐core TFs: *ZEB1*, *ZEB2*, *SNAI1*, *SLUG* and *TWIST1*).^6^, ^25^ Among them, *SLUG* was significantly upregulated or downregulated, respectively, in accordance with the overexpression or knockdown of *LINC00501* (Figure 3A and B, Supplementary Figure S5A). To validate these observations, next generation sequencing was used. *SLUG* was clearly downregulated in the MKN45 cells with *LINC00501* knockdown (Figure 3C). Moreover, significant correlation was observed between *LINC00501* and *SLUG* in Cancer Cell Line Encyclopedia database (Figure 3D). Therefore, we concluded that *LINC00501* regulated EMT process through *SLUG*. Further, a series of rescue experiments were performed. Western blot analysis revealed that silencing *SLUG* counteracted the EMT phenotype in AGS‐*LINC00501* cells (Figure 3E, Supplementary Figure S5B). Transwell invasion assays indicated restoration of invasion ability in AGS‐*LINC00501* cells with *SLUG* knockdown compared with AGS‐*LINC00501* cells (Figure 3F). In wound‐healing and transwell assay, *SLUG* knockdown rescued the migration and invasion ability enhanced by *LINC00501* (Figure 3G and H). Collectively, these data provided evidence that *SLUG* played an essential role in *LINC00501*‐induced EMT.

### 3.4 *LINC00501* transactivates transcription of *SLUG*

**FIGURE 4:**
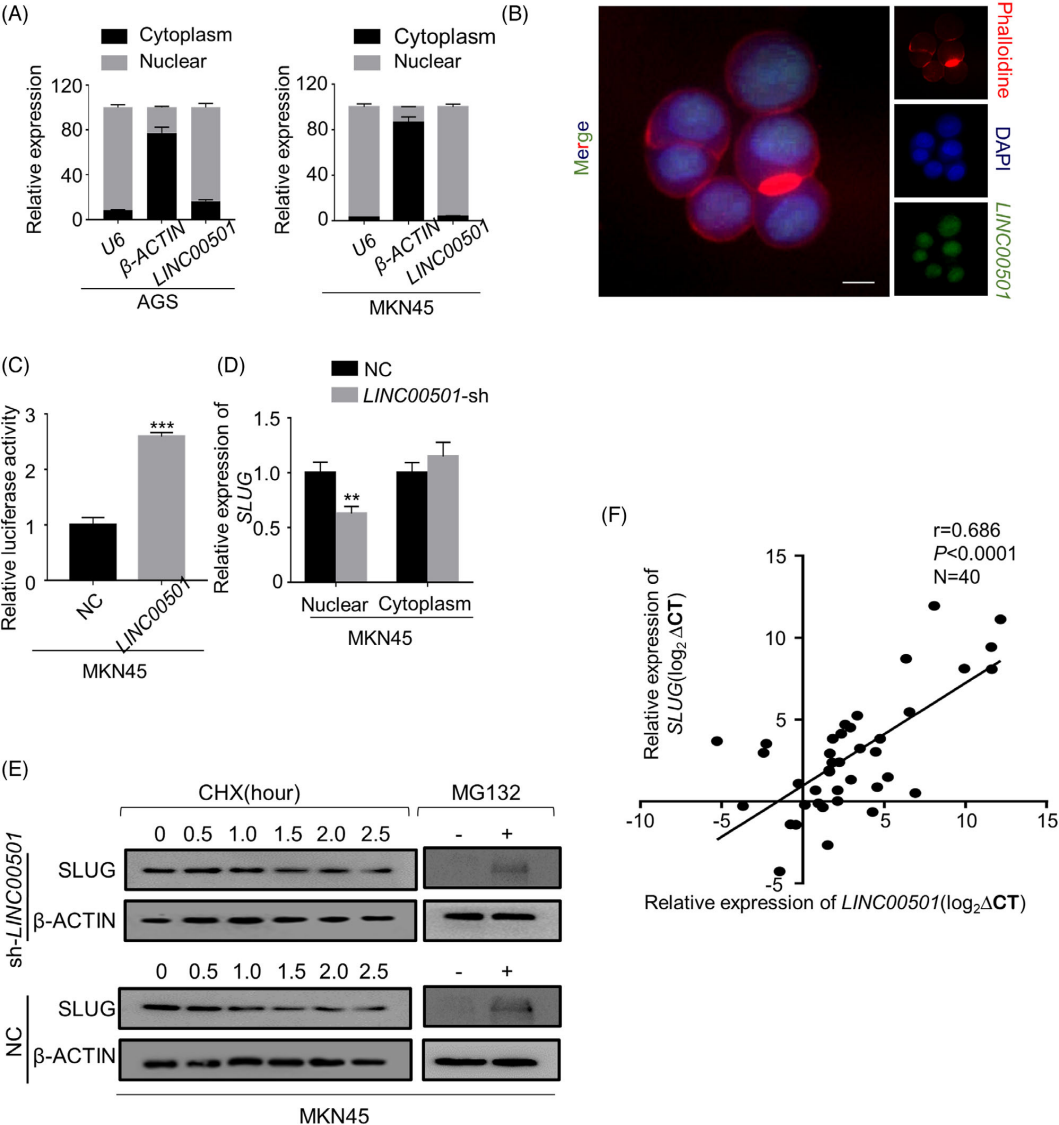
*LINC00501* transactivates transcription of *SLUG*. (A) Subcellular fractionation analyses of *LINC00501* in AGS and MKN45 cell lines. U6 and β‐ACTIN were used as nuclear and cytoplasmic markers. (B) RNA FISH was conducted to study the cellular distribution of *LINC00501*. Scale bar, 10 μm. (C) Dual‐luciferase reporter assays in MKN45 cells cotransfected with the *SLUG* promoter/*LINC00501* plasmids for 48 h. (D) Subcellular fractionation analyses of *SLUG* in MKN45 cell lines after the gain and loss of *LINC00501*. (E) The steady‐state SLUG mRNA levels at 0, 2, 4, and 8 h were quantified using RT‐qPCR after actinomycin D treatment. (F) The correlation analysis between *LINC00501* and *SLUG* was conducted in GC tissues (*n* = 40).

Next, we investigated the regulation mechanism of *SLUG* by *LINC00501*. The functional mechanisms of lncRNAs mainly depend on their subcellular location.^26^ An online predictive tool (https://lncatlas.crg.eu/) revealed that *LINC00501* was mainly located in the nucleus of cells. Subcellular fractionation analyses and FISH assays further confirmed these observations (Figure 4A and B, Supplementary Figure S6A). First, we hypothesised that *LINC00501* regulates SLUG by affecting nearby genes (*TBL1XR*, *KCNMB2*, *ZMAT3*, *PIK3CA* and *KCNMB3*). Indeed, the qRT‐PCR results indicated that *LINC00501* knockdown resulted in a moderate upregulation of *ZMAT3* (approximately 50%), but it did not affect other nearby genes (Supplementary Figure S6B). Furthermore, *ZMAT3* knockdown with corresponding siRNAs did not affect the *SLUG* expression level in AGS cells overexpressing LINC0501 (Supplementary Figure S6C). These results suggested that *LINC00501* transactivated *SLUG* transcription. Since *LINC00501* was enriched in the nucleus, a dual‐luciferase report plasmid which containing the promoter of *SLUG* [–2000‐bp transcription start site (TSS)] was constructed (Supplementary Figure S6D) and cotransfected into MKN45 cell line with *LINC00501* overexpression plasmid. After 48 h of the transfection, the luciferase activity was measured. The promoter activity of *SLUG* was significantly increased by *LINC00501* (Figure 4C). Accordingly, subcellular fractionation analyses revealed that *LINC00501* overexpression increased and *LINC00501* knockdown decreased the component of *SLUG* in nucleus (Figure 4D). Further, other potential posttranscriptional regulations by *LINC00501* were investigated. First, GC cell lines were treated with 10 μM cycloheximide (CHX) and proteasome inhibitor MG132 (10 μM) to block the internal protein synthesis and degradation activity, respectively. SLUG protein level was detected using western blot analysis. *LINC00501* had no obvious effect on the stability of SLUG protein (Figure 4E). In the end, the examination of 40 sets of GC tissues using qRT‐PCR demonstrated a noteworthy and affirmative association between *LINC00501* and *SLUG* (*R* = 0.686, *p* < .0001; Figure 4F). The findings suggested that *LINC00501* enhanced *SLUG* transcription.

### 3.5 *LINC00501* interacts with hnRNPR and enhances *SLUG* expression

**FIGURE 5:**
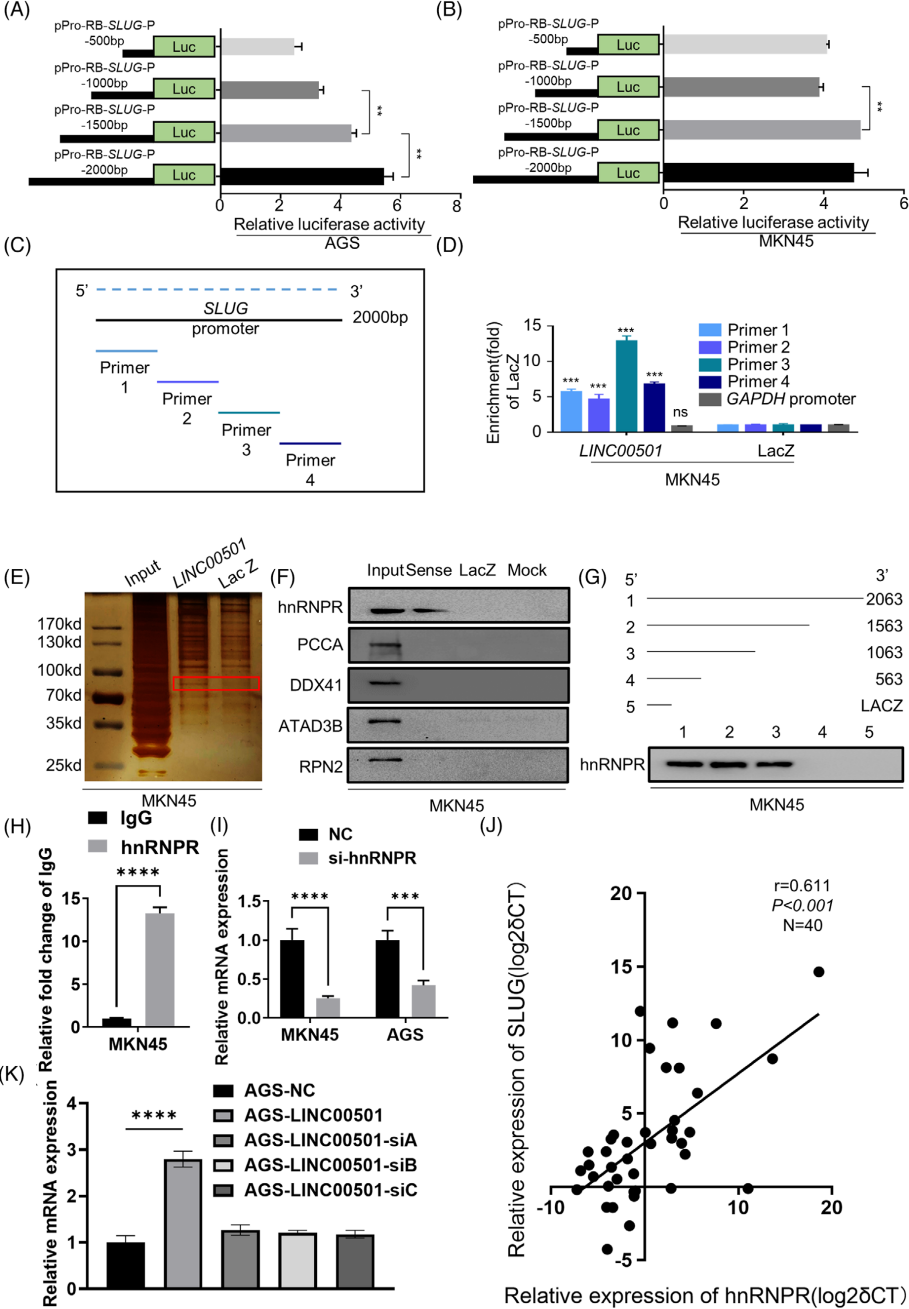
*LINC00501* interacts with hnRNPR and enhances *SLUG* expression. (A) Luciferase assay was performed to evaluate the activity of the *SLUG* promoter using the transfection of truncated or full‐length *SLUG* promoter reported vectors in AGS cells. (B) Luciferase assay was performed to evaluate the activity of the *SLUG* promoter using the transfection of truncated or full‐length *SLUG* promoter reported vectors in MKN45 cells. (C) Schematic representation of the primers for chromatin isolation by RNA purification (ChIRP) to explore the binding of *LINC00501* and *SLUG* promoter. (D) Results of ChIRP assays using *LINC00501* or LacZ (NC) antisense probe sets. Promoter of GAPDH served as a NC region. (E) Silver staining of biotinylated *LINC00501*‐interacted proteins. *LINC00501*‐specific bands (marked) were excised and further analysed using mass spectrometry. (F) Western blot analysis of proteins from *LINC00501* and LacZ probe pulldown assays in MKN45 cells. (G) Truncation of *LINC00501* was used to map the core regions of *LINC00501*‐hnRNPR interaction. (H) RNA immunoprecipitation (RIP) assay using the anti‐hnRNPR antibody followed by qRT‐PCR were performed to validate the interaction between hnRNPR and *LINC00501*. (I) qRT‐PCR analysis of *SLUG* expression in hnRNPR‐silenced MKN45 and AGS cell lines. (J) The correlation analysis was conducted between *hnRNPR* and *SLUG* in GC tissues (*n* = 40). (K) qRT‐PCR analysis of *SLUG* expression in hnRNPR‐silenced siRNA transfected AGS‐*LINC00501* cells.

To further assess the molecular mechanism by which *LINC00501* activates *SLUG* transcription, a series of *SLUG* promoter (–2000‐bp TSS) luciferase reporter plasmids were ab initio constructed (Figure 5A and B). The luciferase activity was significantly increased from 1000 to 2000 bp in AGS cells and increased from −1000 to 1500 bp in MKN45 cells. In addition, we conducted a ChIRP assay using a biotinylated probe for *LINC00501* to isolate chromatin and purify RNA in MKN45 cells. This was done to determine the locations where *LINC00501* binds to the *SLUG* promoter. *LINC00501* probe bound to the whole genome area ranging from −2000 bp to TSS of SLUG promoter compared with LacZ probe (Figure 5C and D). Subsequently, RNA pulldown assay with biotinylated *LINC00501* probe and negative LacZ probe was performed in MKN45 cells. A clear band was observed between 70 and 80 kd in the *LINC00501*‐interacting proteins (Figure 5E). Further, RNA pulldown western blot assay indicated hnRNPR, rather than other proteins in this band, interacted with *LINC00501* (Figure 5F, Supplementary Figure S7A). Serial 5ʹ‐end truncated *LINC00501* probe identified that 563–1063 nt was the essential fragment for the *LINC00501*‐hnRNPR interaction (Figure 5G). RNA immunoprecipitation assay was performed to confirm the interaction between *LINC00501* and hnRNPR. Compared with IgG, marked enrichment of *LINC00501* was observed in the hnRNPR immunoprecipitation group (Figure 5H). hnRNPR is reported promoted metastasis in GC.^27^ Additionally, qRT‐PCR examination revealed a significant upregulation of hnRNPR in gastric cancer tissues (*n* = 40, Supplementary Figure S7B and C). Immunofluorescence was used to investigate the subcellular location of hnRNPR. hnRNPR was mainly located in the nucleus of MKN45 cells (Supplementary Figure S7D). To explore the role of hnRNPR in the regulation of *SLUG*, siRNAs of *hnRNPR* were synthesised and transfected into AGS and MKN45 cells. qRT‐PCR revealed that the expression level of *SLUG* significantly diminished (Figure 5I, Supplementary Figure S7E and F). Further analysis in 40 pairs of GC tissues revealed a positive correlation between *hnRNPR* and *SLUG* (Figure 5J). Knockdown of *hnRNPR* recapitulated the increased *SLUG* mRNA level in AGS‐*LINC00501* cell line, the result indicated downregulation of SLUG and reversed‐EMT phenotype in AGS‐*LINC00501* cell line (Figure 5K). In summary, these results indicated that *LINC00501* regulates *SLUG* through interaction with hnRNPR in GC cells.

### 3.6 *LINC00501* promotes tumour angiogenesis through SLUG/VEGFA/CXCL12

**FIGURE 6:**
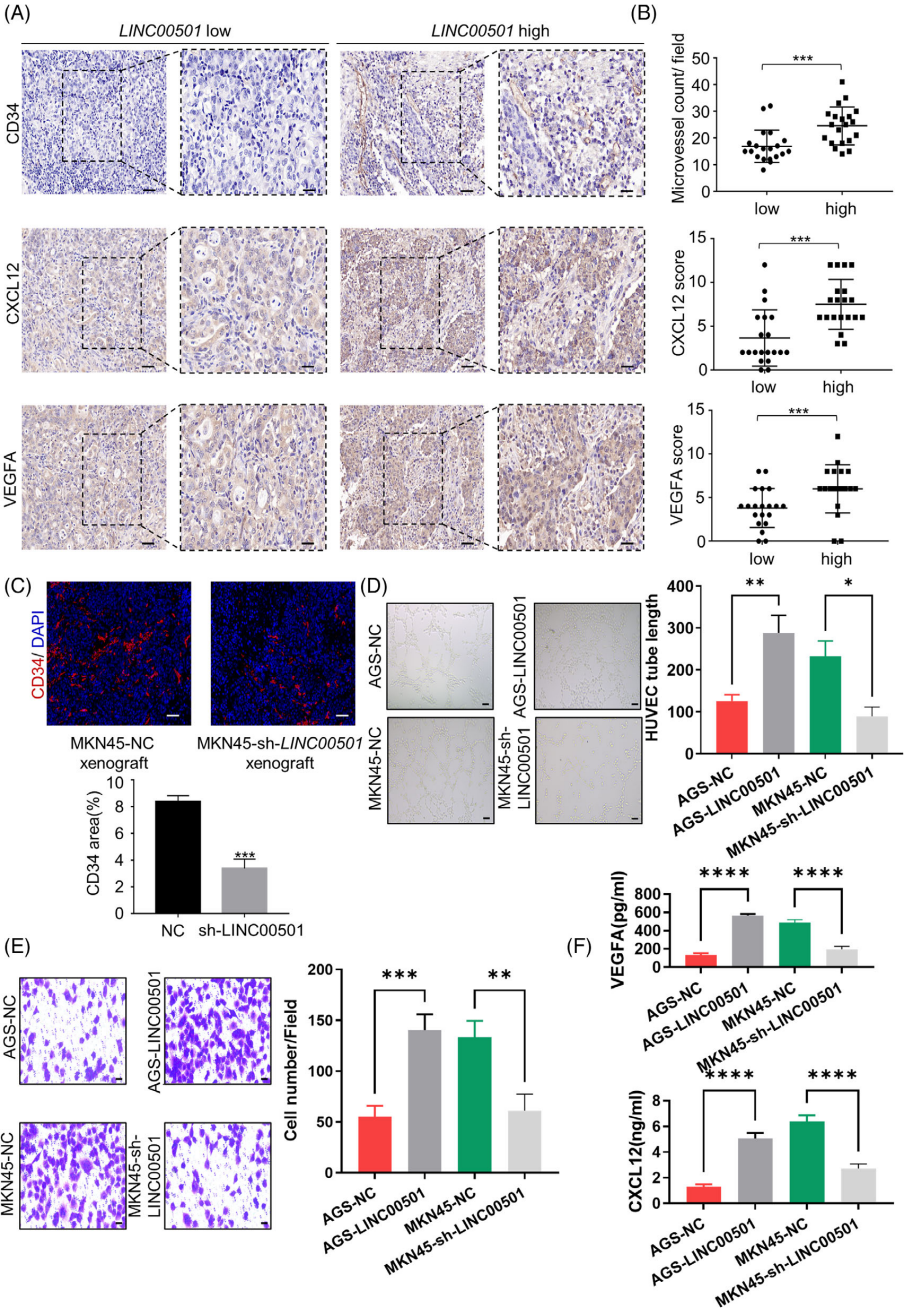
*LINC00501* promotes tumour angiogenesis through SLUG/VEGFA/CXCL12. (A) Representative immunohistochemistry (IHC) staining for CD34/VEGFA/CXCL12 expression in GC tissues with high/low *LINC00501* level. Scale bar, 100 μm. (B) The microvessel count and IHC score of VEGFA/CXCL12 in GC tissues with high/low *LINC00501* level. (C) CD34 expression in mouse GC xenograft model injected with MKN45‐NC/sh‐*LINC00501* cells. Tube formation assay (D) and transwell migration assay (E) (magnificence: 200×) of HUVECs treated with the conditioned medium from indicated AGS cells and MKN45 cells. Scale bar for tube formation assay, 100 μm. Scale bar for migration assay, 20 μm. (F) ELISA assay of VEGFA/CXCL12 protein secretion of AGS‐NC/AGS‐*LINC00501* and MKN45‐NC/MKN45‐sh‐*LINC00501*.

It is well established that EMT process is associated with tumour angiogenesis,^11^ and SLUG expressed in tumour cells promotes the angiogenesis through VEGFA and CXCL12 production.^28^, ^29^ We further evaluated the blood vessel density and VEGFA and CXCL12 levels in GC tissues. Tumour tissues exhibiting high *LINC00501* expression showed a notable increase in the number of blood vessels. Similarly, the levels of VEGFA and CXCL12 IHC scores showed an increase in GC tissues with elevated *LINC00501* expression (Figure 6A and B). A CD34 antibody was used to mark the blood vessels in the xenografts from the MKN45‐NC and MKN45‐sh‐*LINC00501* groups. The results revealed that *LINC00501* positively correlated with the expression of vascular endothelial marker CD34 and blood vessel generation (Figure 6C). To assess the angiogenesis‐promoting effect of *LINC00501* in vitro, HUVEC cells were cultured with the conditioned medium from AGS‐*LINC00501*/NC cells, and subsequently, the tube formation and transwell assays were performed on of HUVEC cells. The conditioned medium from AGS cells overexpressing *LINC00501* significantly enhanced the tube formation and migration abilities, and the knockdown of *LINC00501 exhibited opposite* effects (Figure 6D and E). In addition, the level of VEGFA and CXCL12 in the conditioned medium from AGS‐NC/AGS‐*LINC00501* and MKN45‐NC/MKN45‐sh‐*LINC00501* was measured using enzyme‐linked immunosorbent assay. The concentration of VEGFA/CXCL12 in the cultured medium of AGS‐*LINC00501* significantly rose, while the depletion of *LINC00501* had a contrary impact on the level of VEGFA/CXCL12 (Figure 6F). Collectively, these results suggested that *LINC00501*/SLUG axis promoted angiogenesis through VEGFA and CXCL12 production.

### 3.7 The upstream of *LINC00501* and potential pharmacological intervention

**FIGURE 7:**
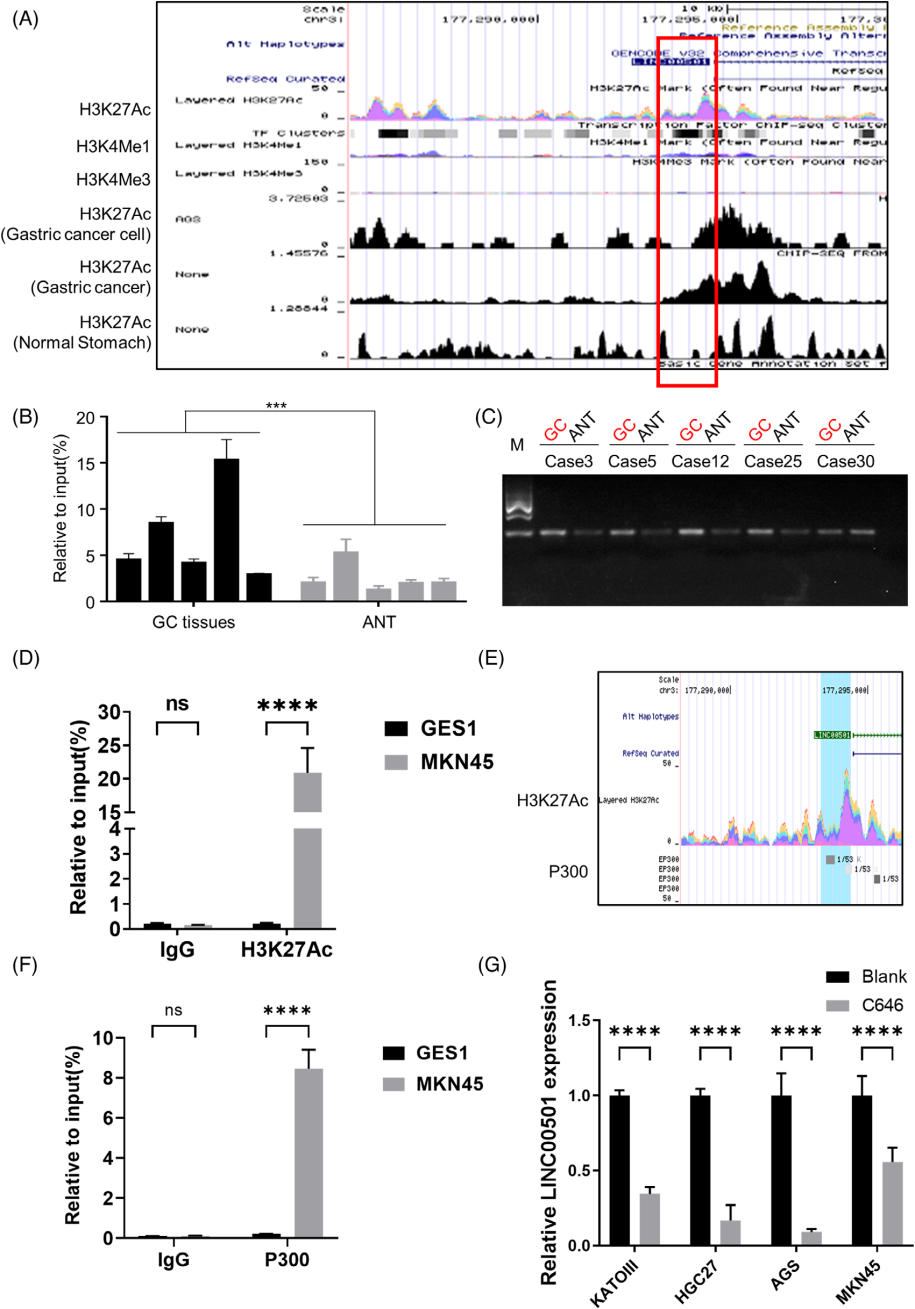
*LINC00501* is activated by H3K27 acetylation in GC. (A) The genome bioinformatics sites, namely, UCSC (http://genome.ucsc.edu/) and Cistrome (http://cistrome.org/db/#/) showed a high enrichment of H3K27 acetylation in the promoter region of *LINC00501*. (B, C) ChIP assays followed by qRT‐PCR revealed the enrichment level of H3K27ac in the promoter region of *LINC00501* in 5 pairs of GC tissues. (D) ChIP assays followed by qRT‐PCR revealed the enrichment level of H3K27ac in the promoter region of *LINC00501* in normal gastric epithelial cell line GES1 and MKN45 cells. (E) Potential binding of P300 in the promoter region of *LINC00501* was analysed using UCSC (http://genome.ucsc.edu/). (F) ChIP assays followed by qRT‐PCR revealed the enrichment level of P300 in the promoter region of *LINC00501* in normal gastric epithelial cell line GES1 and MKN45 cells. (G) Four GC cell lines were treated with P300 inhibitor (C646). qRT‐PCR was performed to analyse the expression level of *LINC00501*.

To understand the underlying mechanism of *LINC00501* upregulation in GC tissues, we first used two bioinformatics websites, UCSC genome browser and Cistrome, to analyse the promoter region of *LINC00501*. Notably, a significant abundance of H3K27ac was observed at the promoter region of *LINC00501*, particularly in GC tissues when compared to normal tissue (Figure 7A). In order to uncover the genuine H3K27ac enrichment in the promoter region of *LINC00501* in GC, a CHIP assay was conducted on five pairs of GC tissues and their corresponding normal tissues. The findings indicated an increase in H3K27ac levels at the promoter region of *LINC00501*, with a higher enrichment observed in GC tissues in comparison to normal tissues (Figure 7B and C). Consistently, we also found an enrichment of H3K27ac at *LINC00501* promoter in MKN45 cells compared with GES1 (Figure 7D). In addition, at the H3K27ac enrichment peak region of *LINC00501* promoter, a potential P300 bound was detected (Figure 7E).  Given that P300 has been established as a histone acetyltransferase enzyme capable of activating gene transcription, we further conducted a CHIP assay to verify the enrichment of P300, the results showed a significantly bound of P300 in the promoter of *LINC00501* (Figure 7F). On this basis, we conducted CO‐IP assay, and the results showed the interaction between H3K27ac and P300 (Supplementary Figure S8A). Concordantly, administration of C646, a well‐known P300 inhibitor, markedly reduced the expression level of *LINC00501* in GC cell lines (Figure 7G, Supplementary Figure S8B). Additionally, we also evaluate the methylation level by analysing the CpG island in the promoter of *LINC00501* (Methprimer, http://www.urogene.org/methprimer/, Supplementary Figure S8C), which indicated no obvious CpG island in the promoter region. In addition, after lowering the threshold, a target BSP was utilised to assess the methylation level in the promoter of *LINC00501*, the results showed that the DNA methylation level of *LINC00501* promoter in GC cells and GES1 exhibited no obvious difference (Supplementary Figure S8D). On this basis, the online transcription factor prediction analysis was applied to predict the potential binding in LINC00501's promoter to reveal the potential upstream regulation after H3K27ac (Supplementary Figure S8E). These data suggested the upregulation of *LINC00501* mainly attributed to increased H3K27ac enrichment in the promoter region of *LINC00501*.

### 3.8 Therapeutic potential of *LINC00501* in vivo

**FIGURE 8:**
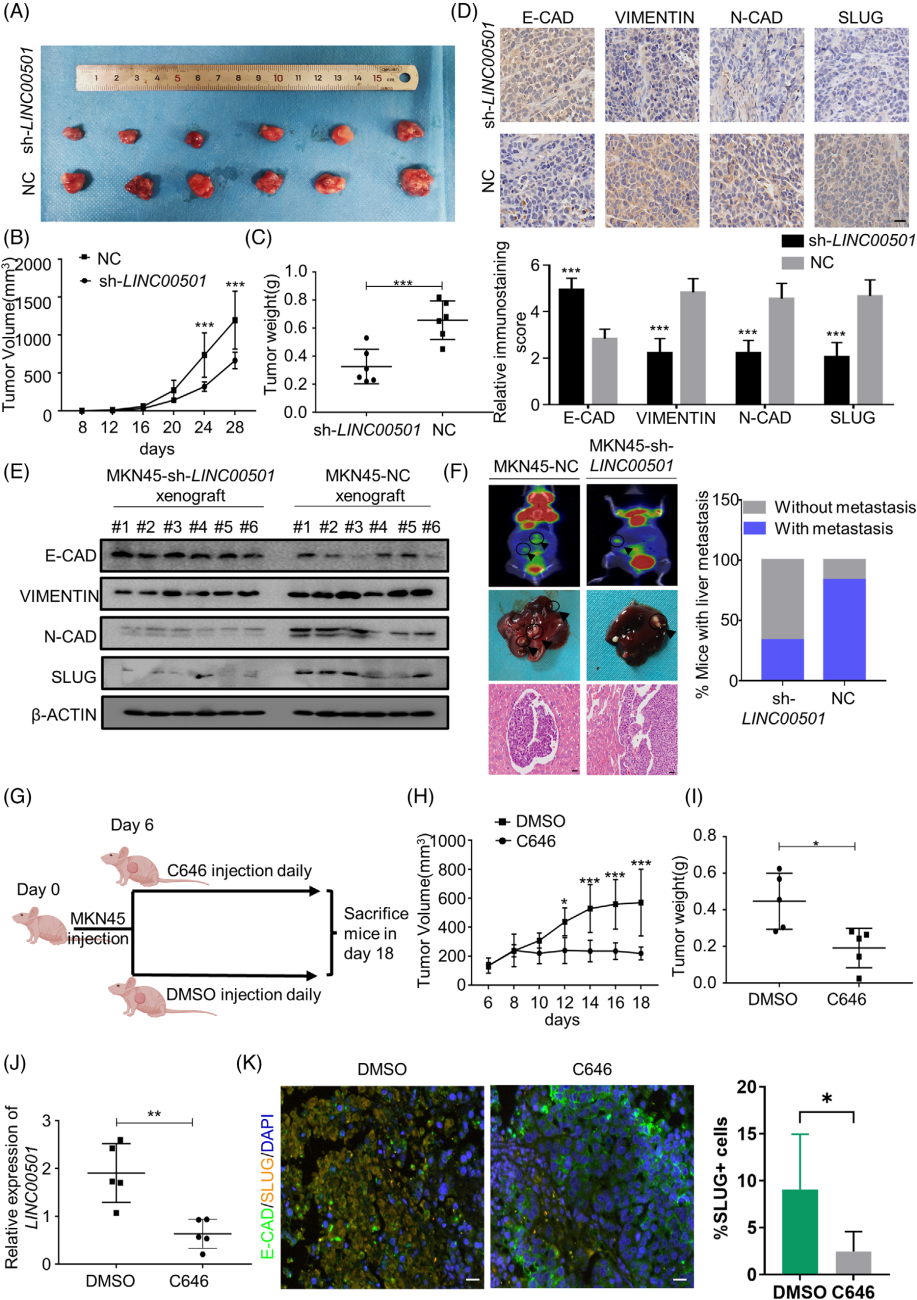
Therapeutic potential of *LINC00501* in vivo. (A) The morphological characteristics of tumour xenografts in the MKN45‐NC and MKN45‐sh‐*LINC00501* groups. (B) Tumour volumes were compared between the MKN45‐NC and MKN45‐sh‐*LINC00501* groups. (C) Tumour weights were compared between the MKN45‐NC and MKN45‐sh‐*LINC00501* groups. (D) Immunohistochemistry and IHC score of E‐CADHERIN, N‐CADHERIN and VIMENTIN in xenograft tumour tissues. Scale bar 50 μm. (E) Western blot analysis of indicated proteins in xenografts produced by MKN45 cells with *LINC00501*‐knockdown and those by MKN45‐NC. (F) Five‐week‐old mice were intrasplenically injected with MKN45‐sh‐*LINC00501*/MKN45‐NC cells. After 20 days, the status of liver metastasis was evaluated. The representative PET‐CT images of nude mice intrasplenically injected with MKN45‐sh‐*LINC00501*/NC cells are given. The percentage of nude mice with or without metastasis in the MKN45‐sh‐*LINC00501*/NC groups was analysed. Scale bar 50 μm. (G) Schematic diagram showing the pharmacological treatment of mice bearing the xenografts derived from MKN45 with C646. (H) Tumour volumes were compared between the C646 and control DMSO groups. (I) Tumour weights were compared between the C646 and control DMSO groups. (J) The expression of *LINC00501* was analysed by qRT‐PCR after C646 treatment. (K) The percentage of SLUG+ cells was analysed in C646 treatment group and control group. Scale bar, 50 μm.

**FIGURE 9:**
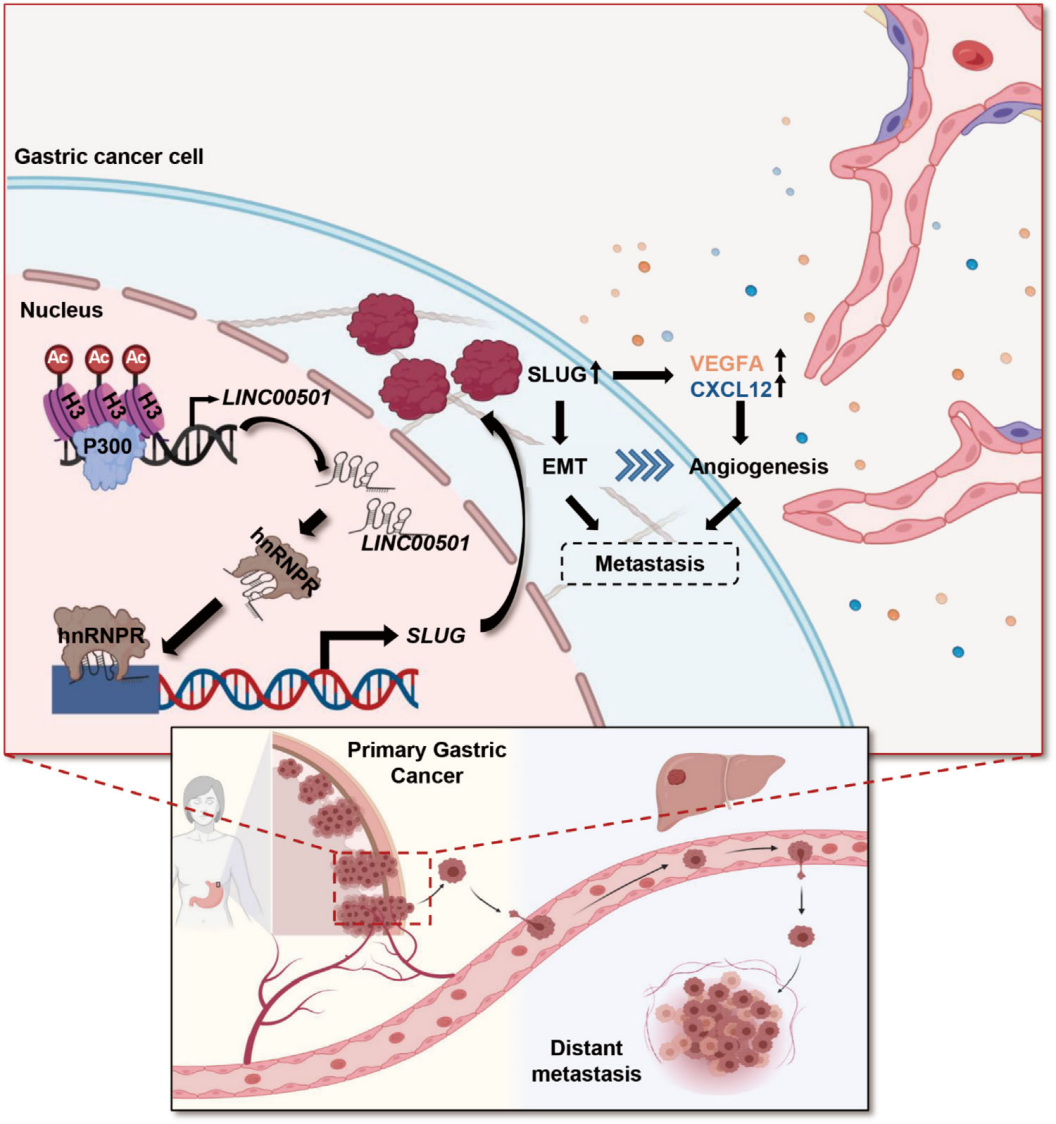
Schematic diagram. *LINC00501*, dysregulated in gastric cancer, promotes tumour invasion and angiogenesis by activating EMT process through hnRNPR/SLUG pathway.

Subsequently, we showcased the impact of *LINC00501* on the initiation of EMT and the spread of tumours in live organisms, while also investigating potential therapeutic approaches aimed at *LINC00501*. Lentivirus targeting *LINC00501* (sh‐*LINC00501*) was applied to knockdown *LINC00501* in MKN45 cell lines. Female nude mice were inoculated with MKN45‐sh‐*LINC00501*/NC cells in the right flank via subcutaneous injection. The xenografts produced from MKN45 cells with *LINC00501* knockdown had lighter weight and smaller volume compared with MKN45‐NC cells (Figure 8A–C). qRT‐PCR revealed that *LINC00501* and *SLUG* levels were significantly higher in MKN45‐NC xenograft (Supplementary Figure S9A and B). Furthermore, *LINC00501* was positively correlated with *SLUG* in xenografts (Supplementary Figure S9C). Immunohistochemistry (IHC) and western blot revealed a downregulated SLUG level in MKN45‐sh‐*LINC00501* cells compared with MKN45‐NC (Figure 8D). Moreover, the levels of EMT markers were analysed, and the results revealed that knockdown of *LINC00501* mitigated EMT phenotype in vivo (Figure 8D and E). In order to further confirm the involvement of *LINC00501* in enhancing metastasis, we created a liver metastasis model in nude mice. The nude mice model was injected intrasplenically with MKN45‐sh‐*LINC00501*/NC cells. Liver metastasis was observed in five out of six nude mice in the MKN45‐NC group. In contrast, only two out of six nude mice in the MKN45‐sh‐*LINC00501* group developed liver metastasis. Furthermore, the MKN‐NC group exhibited a significantly greater quantity of metastatic nodules, as depicted in Figure 8F. Since P300 plays a vital part in the abnormally increased levels of *LINC00501* in GC, we next testify whether the pharmacological intervention of P300 activity with C646 could impact the expression of *LINC00501* and GC tumour growth. As shown in Figure 8G–I, in the preclinical study, administration of C646 significantly reduced the growth of MKN45 gastric cancer cells but without significant loss in body weight (Supplementary Figure S9D). Furthermore, the administration of C646 notably reduced the levels of *LINC00501* and the proportion of SLUG+ cells in xenografts (Figure 8J and K). The combined data indicated that *LINC00501* promoted the process of EMT and metastasis in living organisms, and targeting the expression of *LINC00501* could be a promising therapeutic strategy for GC (Figure 9).

## 4 DISCUSSION

Tumour progression and subsequent metastasis is still the leading cause of death in GC, and currently, no effective treatment options are available.^30^, ^31^ In this study, we identified a lncRNA, namely, *LINC00501*. It was highly expressed in GC tissues and was associated with advanced stage and metastasis of GC. Furthermore, the results of ROC analysis indicated that *LINC00501* may serve as a biomarker for advanced GC. Moreover, patients with high *LINC00501* expression had shorter RFS than those with low *LINC00501* expression. *LINC00501* enhanced migration and invasion of tumour cells and promoted the EMT process by upregulating *SLUG*. The underlying mechanism of action was as follows. *LINC00501* transactivated *SLUG* expression by recruiting RNA‐binding protein hnRNPR to *SLUG* promoter and increased the promoter activity of *SLUG*. We observed that the H3K27ac enrichment in the promoter of *LINC00501* attributed to the upregulation of *LINC00501* in GC. These findings helped in better understanding of lncRNA‐mediated EMT process that promotes the progression and metastasis of GC.

lncRNA is a noncoding RNA with a length >200 nt. Generally, its coding capacity is scarce, which was regarded as nonsense noises in transcripts.^32^, ^33^ However, several studies confirmed that lncRNAs have unique and important biological functions including gene regulation, RNA translocation, RNA stability modulation and translation regulation in tumourigenesis and development of tumours via various mechanisms.^34^, ^35^, ^36^, ^37^ Recent studies proved that RNA‐binding proteins participate in the lncRNA‐mediated gene regulation. Søndergaard et al. reported that CCT3 interacted with *LINC00326* to regulate lipid metabolism in hepatic cancer.^38^ He et al. reported that lncRNA *RBAT1* cis‐activated *E2F3* by binding with hnRNPL and thus promoted tumourigenesis in retinoblastoma.^39^ Yin et al. recently reported that the interaction between YBX1 and lncRNA *RMRP* could subsequently activate *TGFBR1* expression in nonsmall cell lung cancer.^40^ hnRNPs are the key proteins in the nucleic acid metabolism process.^41^ hnRNPR, as a member of hnRNPs, binds to the promoter region to activate transcription.^42^ We observed that *LINC00501* interacted with hnRNPR and recruited hnRNPR to the promoter of *SLUG*, thus enhancing *SLUG* expression. In this study, several experiments were performed to elucidate the potential regulatory mechanism of *LINC00501* in the regulation of *SLUG*; however, future studies are warranted to further explore mechanisms such as direct or indirect epigenetic activation and posttranslational modification. Moreover, it is of great significance to identify the mechanism underlying the dysregulation of lncRNAs in cancers. Similar to protein‐coding genes, lncRNAs are subjected to epigenetic regulation, including histone modification, promoter methylation and so on. Dong et al. reported that the enrichment of H3K27 acetylation in promoter enhanced the expression of lncRNA TINCR and promoted trastuzumab resistance.^43^ Lu et al. reported that the decreased methylation level of the promoter amplified the expression of SNHG12 in glioblastoma.^44^ Our data revealed that rather than methylation, the enrichment of H3K27ac in the promoter region of *LINC00501* contributed to the upregulation of *LINC00501* in GC cell lines and tissues. Furthermore, besides the H3K27Ac, the effect of transcription factor and enhancer after the histone modification is also worth to investigating; the transcription factor binding prediction on the promoter of *LINC00501* revealed that numerous transcription factors may also participate in the regulation of *LINC00501*, including NF‐κB, YY1, GATA2^45^, ^46^, ^47^ which have already been reported to prompt gastric cancer invasion and progression. These results indicated some TFs may take advantage of the H3K27Ac and participated in the regulation of *LINC00501*.

It is well known that EMT process enhances tumour metastasis by increasing tumour cell invasion and migration in various cancers, including GC.^48^ Our previous studies reported that EMT process facilitates the progression and metastasis in cancers.^49^, ^50^ Once EMT is activated, the cell will be characterised by a decline in E‐CADHERIN expression and re‐expression of mesenchymal markers.^6^ EMT is a complex process orchestrated with EMT‐core TFs including ZEB1, ZEB2, SNAI1, SLUG and TWIST1.^25^ Among them, numerous studies reported that SLUG, as a core EMT‐TF, promotes tumour metastasis. Li et al. reported that SLUG increased breast cancer metastasis by enhancing tumour cell invasion and migration.^51^ Recouvreux et al. reported that SLUG contributes to the vitality of tumour cells.^52^ It is well documented that *SLUG* is associated with poor clinical outcomes and increased risk of metastasis.^53^ Moreover, the EMT tumour cells can modulate the tumour microenvironment,^54^ and it is reported that the upregulation of SLUG increases the downstream expression of VEGFA and CXCL12 and promotes the angiogenesis.^28^, ^29^ In this study, we demonstrated that *LINC00501* enhanced EMT process by upregulating *SLUG* transcription, thus promoting GC metastasis. These results provided a novel mechanism underlying the regulation of EMT process by lncRNAs. We must admit that except for the EMT prompting effect of *LINC00501*, other mechanisms of *LINC00501* should also be considered in the future including the potential function from the small portion of cytoplasmic *LINC00501*. One more interesting finding is when we conducted the ISH for *LINC00501*, we observed *LINC00501* is mainly expressed in epithelium cells; however, some lymphocyte exhibited strong positive *LINC00501* expression (data not shown), indicating that *LINC00501* may have different cell distribution in tumour and unrevealed mechanisms should be further studied. We believe, with the advancement and employment of single‐cell lncRNA sequence, we will have a better understanding of *LINC00501* regarding the distribution and comprehensive function.

Emerging studies reported that lncRNA is a potential candidate biomarker for GC.^55^, ^56^ *LINC00501*, as an upregulated oncogenic lncRNA, was reported to distinguish GC from normal stomach tissues.^23^ Consistent with this previous study, we reported that *LINC00501* was significantly upregulated in GC tissues than in normal stomach tissues. Importantly, *LINC00501* exhibited the further potential to identify advanced and metastatic GC; the AUCs of the ROC curve were 0.767 and 0.737, respectively. Recently, specific noncoding‐RNA‐targeted agents have been designed as candidates for antitumour therapy.^57^, ^58^ Our study revealed that the knockdown of *LINC00501* with *LINC00501*‐specific lentivirus repressed the invasion and migration of tumour cells. Furthermore, P300 as a transcriptional coactivator and histone acetyltransferases has been proved to involve in several pathological processes, and targeting P300 and thereby inhibiting subsequent downstream oncogenes is emerging as an efficient therapy intervention in several cancers including prostate cancer,^59^ hepatic cancer,^60^ cervical cancer,^61^ glioblastoma^62^ and ovarian cancer.^63^ In our study, we demonstrated that disruption of the P300/*LINC00501* axis by C646 inhibits the tumour growth of GC without significant sign of side effect, indicating that the P300/*LINC00501* axis as a therapeutic target in GC.

## 5 CONCLUSIONS

Our study revealed that LINC00501 is highly expressed in GC and promotes GC metastasis by enhancing the EMT process and tumour environment remodel. To describe the underlying mechanism, LINC00501 promoted SLUG expression through hnRNPR. Understanding the precise role of *LINC00501*/hnRNPR/*SLUG* regulatory axis in advanced GC will not only expand our knowledge on the underlying mechanism of progression and metastasis of GC but also help to develop a promising biomarker and potential therapeutic target in GC.

## FUNDING INFORMATION

We appreciate the funds from National Natural Science Fund Youth Fund of China (81702411); National Natural Science Foundation of China (82173330); the mainstay project of young and middle‐aged medicine in Wuhan (WHQG202003); and Zhongnan Hospital of Wuhan University, Technology Innovation Seed Found (znpy2019076).

## CONFLICT OF INTEREST STATEMENT

No conflicts to declaration.

## Supporting information

Supporting Information

## Data Availability

The sequencing data of RNA produced in this research can be found in the GEO database GSE193109. Additional experimental data and resources can be found on the website of Clinical and Translational Medicine.
