# Supplementary material for: Upregulation of LINC00501 by H3K27 acetylation facilitates gastric cancer metastasis through activating epithelial‐mesenchymal transition and angiogenesis
Source: Clin Transl Med. 2023 Oct 23;13(10):e1432. doi: 10.1002/ctm2.1432 (PMC10591115; doi:10.1002/ctm2.1432)
Supplement: Supplementary file 1 — Supporting Information [file CTM2-13-e1432-s001.docx]

Supplementary material for

**Upregulation of *LINC00501* by H3K27ac facilitates gastric cancer metastasis through activating** **epithelial-mesenchymal transition and angiogenesis**

Rongzhang Dou^1,2,3,4*^, Lei Han^1,2,3,4*^, Chaogang Yang^1,2,3,4*^, Yan Fang^5*^, Jinsen Zheng^1,2,3,4^, Chenxi Liang^1,2,3,4^, Jialin Song^1,2,3,4^, Chen Wei^6^, Guoquan Huang^1,2,3,4^, Panyi Zhong^1,2,3,4^, Keshu Liu^1,2,3,4^, Qian Peng^7^, Chunwei Peng^1,2,3,4,^ Bin Xiong^1,2,3,4^ and Shuyi Wang^1,2,3,4^

1. Department of Gastrointestinal Surgery, Zhongnan Hospital of Wuhan University, No.169 Donghu Road, Wuhan, Hubei, 430071, China.

2. Department of Gastric and Colorectal Surgical Oncology, Zhongnan Hospital of Wuhan University, No.169 Donghu Road, Wuhan, Hubei, 430071, China.

3. Hubei Key Laboratory of Tumor Biological Behaviors, No.169 Donghu Road, Wuhan, Hubei, 430071, China.

4. Hubei Cancer Clinical Study Center, No.169 Donghu Road, Wuhan, Hubei, 430071, China.

5. Department of Pathology, Zhongnan Hospital of Wuhan University, No.169 Donghu Road, Wuhan, Hubei, 430071, China.

6. Department of Internal Medicine, Affiliated Tumor Hospital of Zhengzhou University, Henan Cancer Hospital, Zhengzhou, 450008, China

7. Guangzhou Women and Children’s Medical Center

Correspondence: Dr. Shuyi Wang, E-mail: shuyiwang@whu.edu.cn

Correspondence: Dr. Bin Xiong, E-mail: binxiong1961@whu.edu.cn

Correspondence: Dr. Chunwei Peng, E-mail: whupengcw@whu.edu.cn

*These authors contributed equally to this work.

**Supplementary Figure legends**

**Supplementary Figure 1 Microarray analysis for 6 pairs of GC tissues**

1. Schematic process to screen the lncRNA LINC00501.

**Supplementary Figure 2 Basic characteristics of *LINC00501***

1. The expression of *LINC00501* in various gastrointestinal tumors.
2. The bioinformatics prediction to the coding potential of *LINC00501* (https://lncar.renlab.org/).
3. The secondary structure of *LINC00501* (https://lncar.renlab.org/).

**Supplementary Figure 3 *LINC00501* correlates with advanced GC stage and metastasis**

1. ROC analysis was conducted to show the ability of *LINC00501* expression in distinguishing gastric cancer patients by using TCGA dataset (n=407).
2. ROC analysis was conducted to show the ability of *LINC00501* expression in distinguishing high stage (Ⅲ+Ⅳ) from low stage (Ⅰ+Ⅱ) patients in cohort2 (n=304).
3. ROC analysis was conducted to show the ability of *LINC00501* expression in distinguishing metastasis (M1) from non-metastasis (M0) patients in cohort2 (n=304).
4. Overall survival was analyzed and compared between patients with high and low levels of *LINC00501* expression in GC patients in cohort 2 (n=304).

**Supplementary Figure 4 The correlation between *LINC00501* and EMT and the construction of stable-transfected cell lines**

1. The expression of LINC00501 was testified by qRT-PCR in several gastric cancer cell lines.
2. qRT-PCR analysis to testify the overexpressed-transfection efficiency of *LINC00501* in AGS cells.
3. qRT-PCR analysis to testify the knockdown-transfection efficiency of *LINC00501* in MKN45 cells.

**Supplementary Figure 5 SLUG is required for *LINC00501*-induced EMT**

1. Western blot for EMT core TFs in indicated cell lines was quantified.
2. Western blot for EMT core markers in indicated cell lines was quantified.

**Supplementary Figure 6 *LINC00501* upregulates SLUG in a trans-transcriptional manner**

1. The online prediction (https://lncatlas.crg.eu/) of the subcellular distribution of *LINC00501*.
2. The nearby genes regulated by *LINC00501*. (left panel)The chromatin location of *LINC00501* (NCBI, https://www.ncbi.nlm.nih.gov/gene/?term=*LINC00501*). (right panel) qRT-PCR analysis of nearby gene of *LINC00501* in *LINC00501* knocked down GC cells.
3. Western blot analysis of SLUG in AGS-*LINC00501* cells with/without combination ZMAT3 knockdown.
4. The schematic of SLUG promoter plasmid (pPro-RB-report).

**Supplementary Figure 7 *LINC00501* interact with hnRNPR and activate *SLUG* expression**

1. The online prediction (RPISeq, http://pridb.gdcb.iastate.edu/RPISeq/) of *LINC00501*-hnRNPR interaction.
2. The expression of hnRNPR in various gastrointestinal tumors.
3. qRT-PCR analysis of *hnRNPR* expression level in 40 pairs GC tissues and matched NATs.
4. The sub-cellular location of hnRNPR in MKN45 cells. Scale bar, 20 μm
5. qRT-PCR analysis to testify the knockdown efficiency of hnRNPR in MKN45 cells.
6. Western blot analysis of hnRNPR after knockdown with hnRNPR-siRNAs in MKN45 cells.

**Supplementary Figure 8 DNA methylation is not involved in the activation of *LINC00501***

1. Co-immunoprecipitation assay was conducted to determine the interaction between P300 and H3K27ac
2. Dual-luciferase reporter assays in MKN45 and AGS cells transfected with the LINC00501 promoter with/without C646 treatment for 48 h.
3. Schematic representation of the CpG islands (Methprimer, http://www.urogene.org/methprimer/ )
4. Individual methylation level of each CpG cytosine within the *LINC00501* promoter.
5. The prediction binding of TFs in the promoter of LINC00501 based on online website PROMO (https://alggen.lsi.upc.es/cgi-bin/promo_v3/promo/promoinit.cgi?dirDB=TF_8.3).

**Supplementary Figure 9 Therapeutic potential of *LINC00501* *in vivo***

1. qRT-PCR was conducted to analyze the expression of *LINC00501* in MKN45-sh-*LINC00501*/NC xenografts.
2. qRT-PCR was performed to analyze the expression of *SLUG* in MKN45-sh-*LINC00501*/NC xenografts.
3. The correlation between *LINC00501* and *SLUG* in xenografts was analyzed.
4. The morphological characteristics of tumor xenografts in the in MKN45 xenografts groups treated with/without C646 .
5. Mouse weight of after treatment of C646 in MKN45 xenografts.


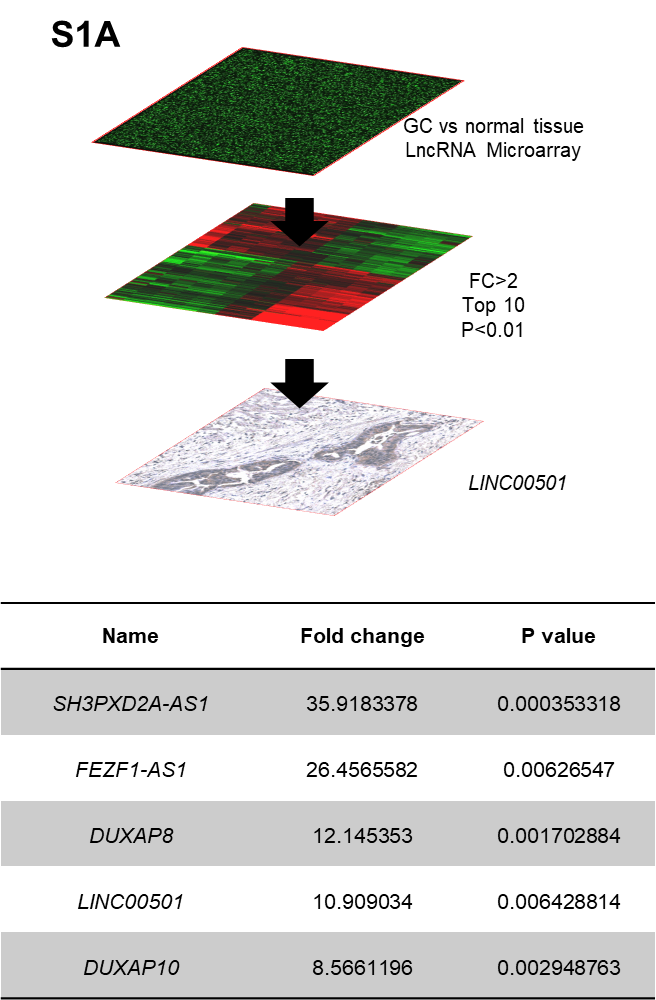


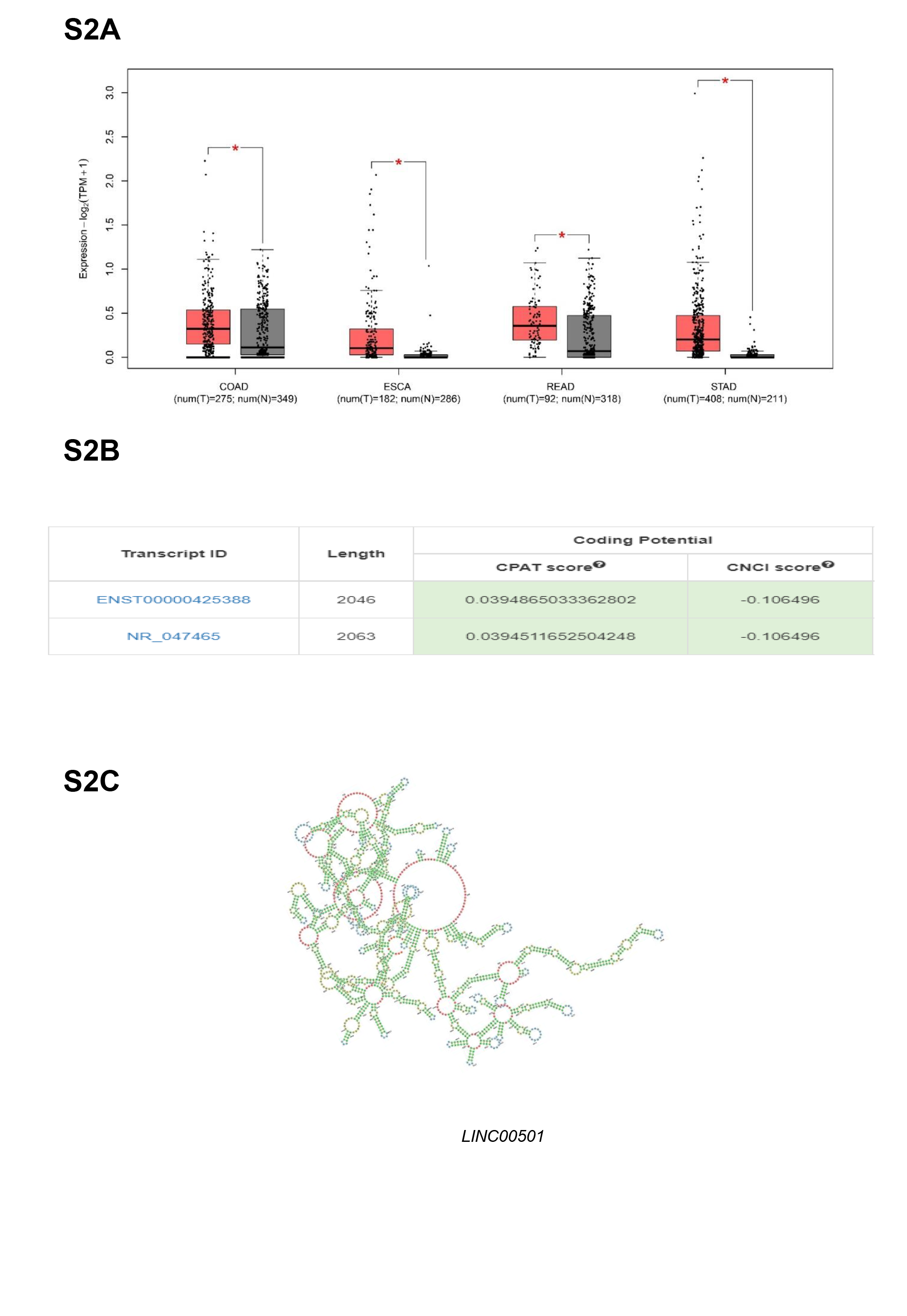


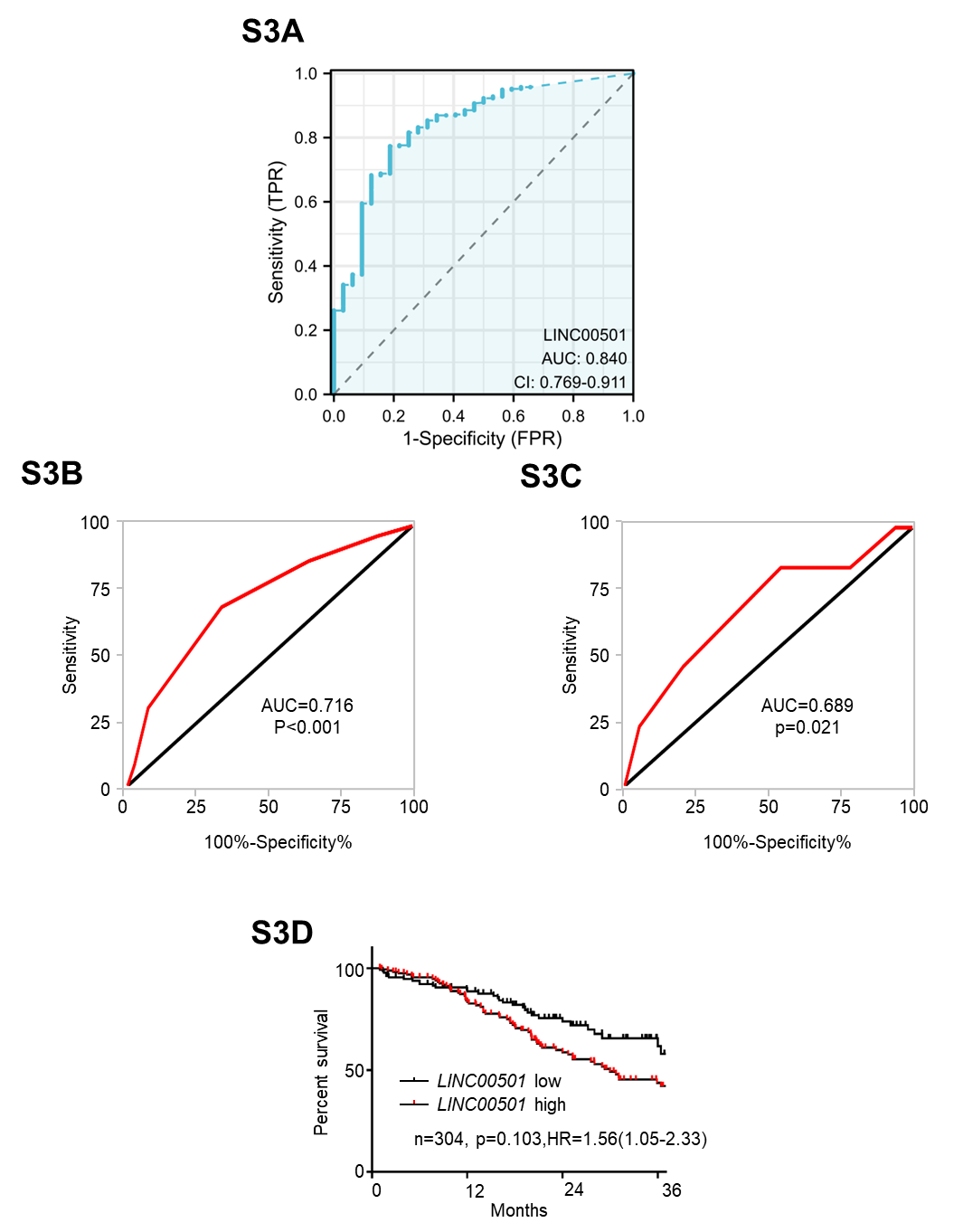


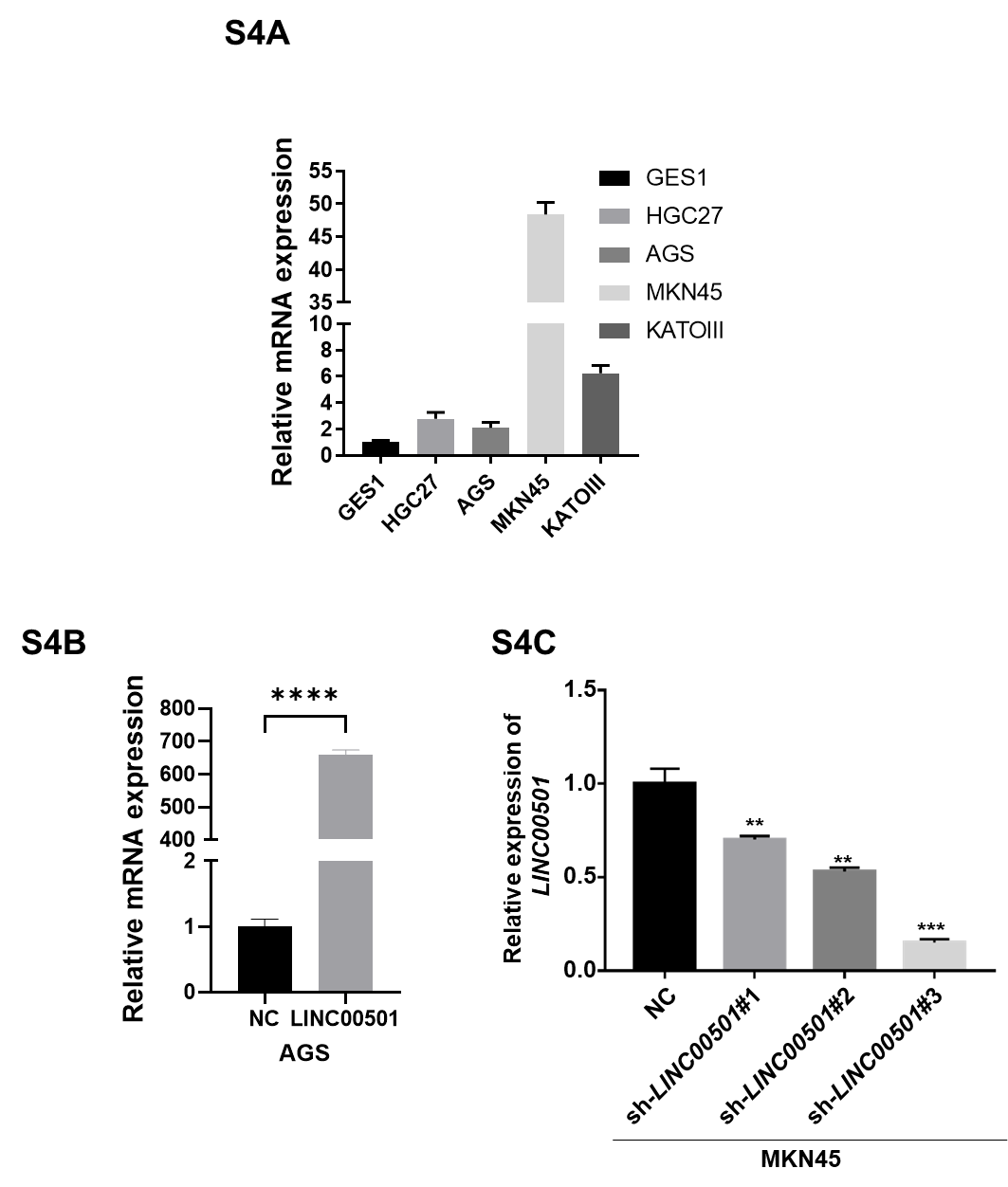


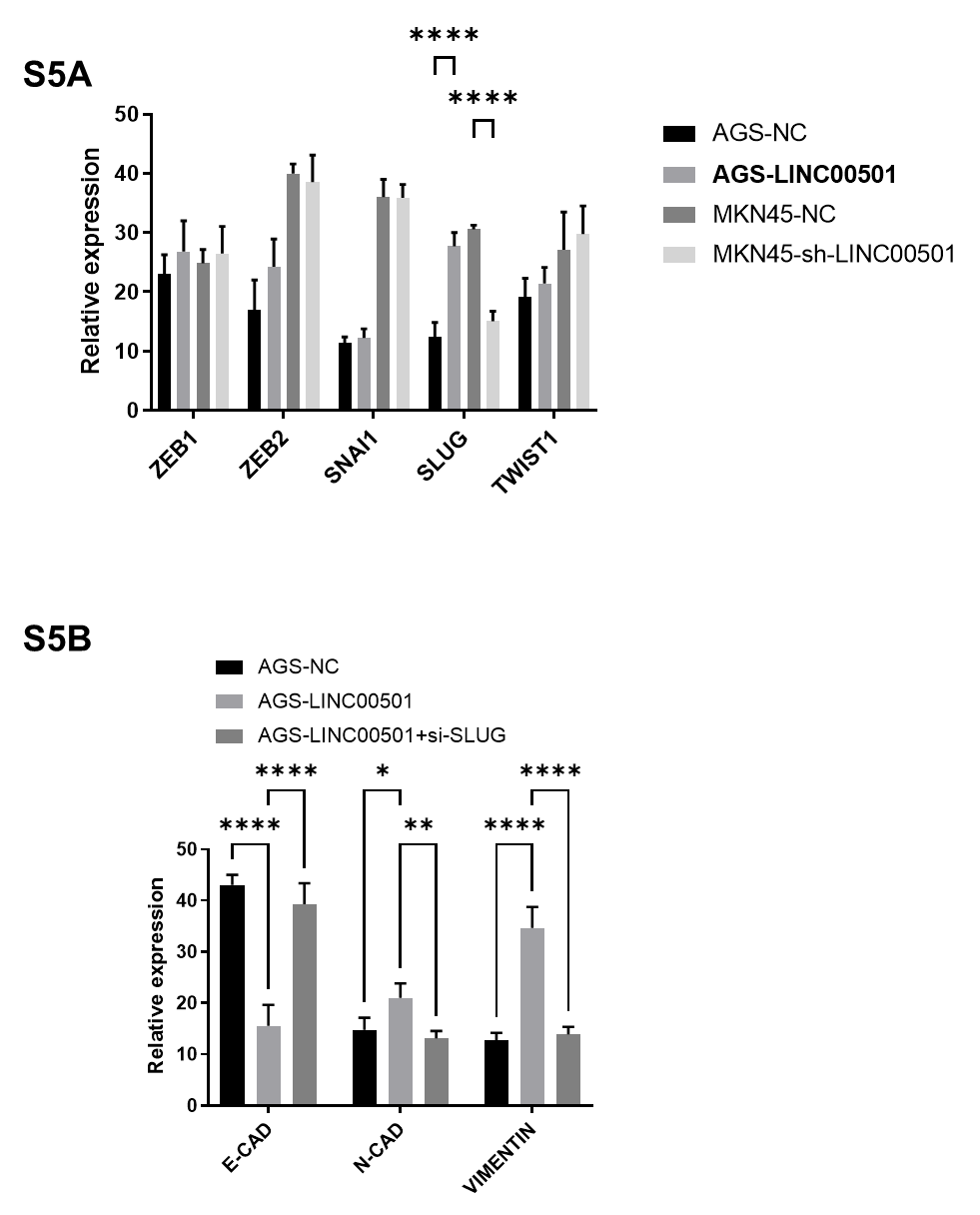


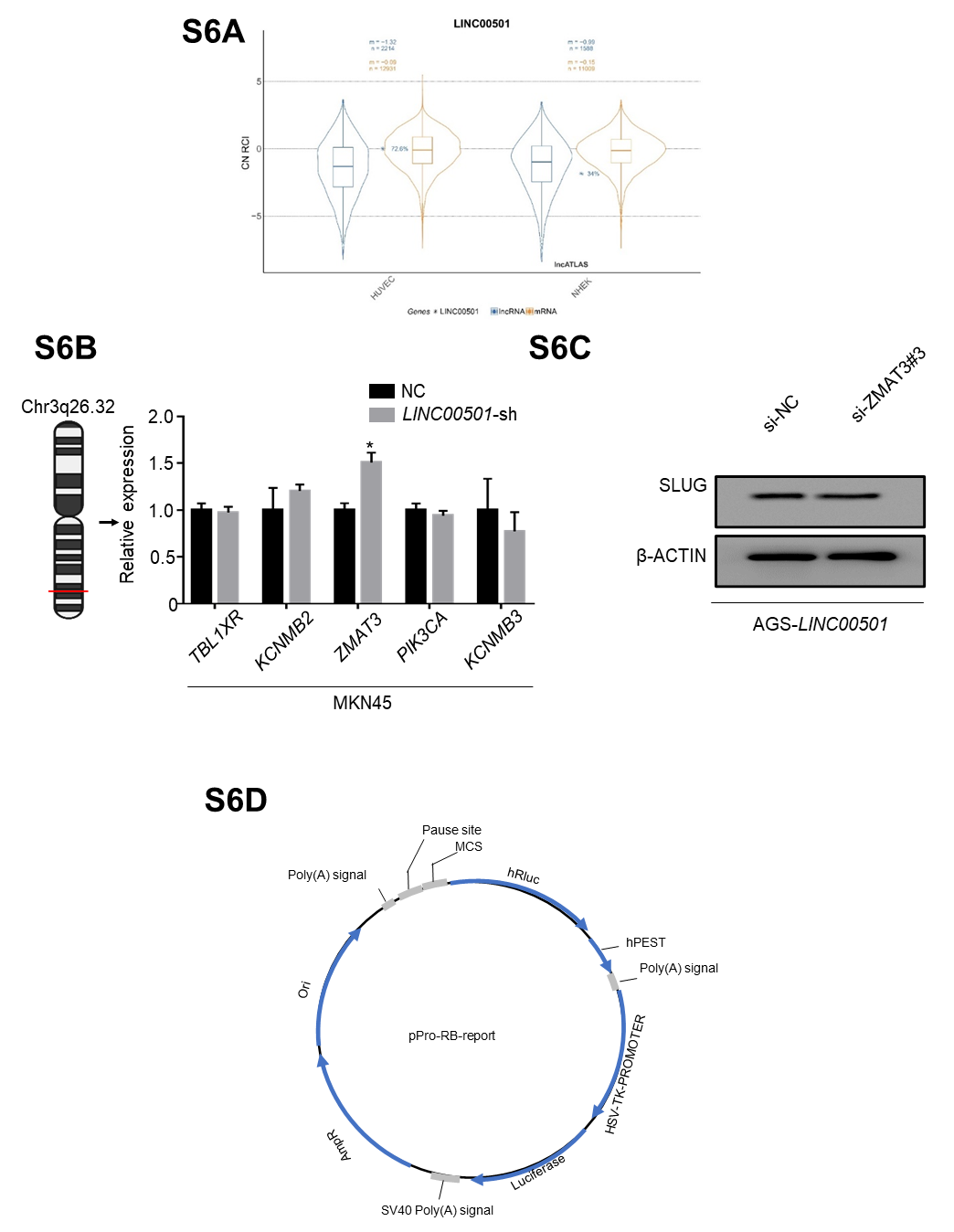


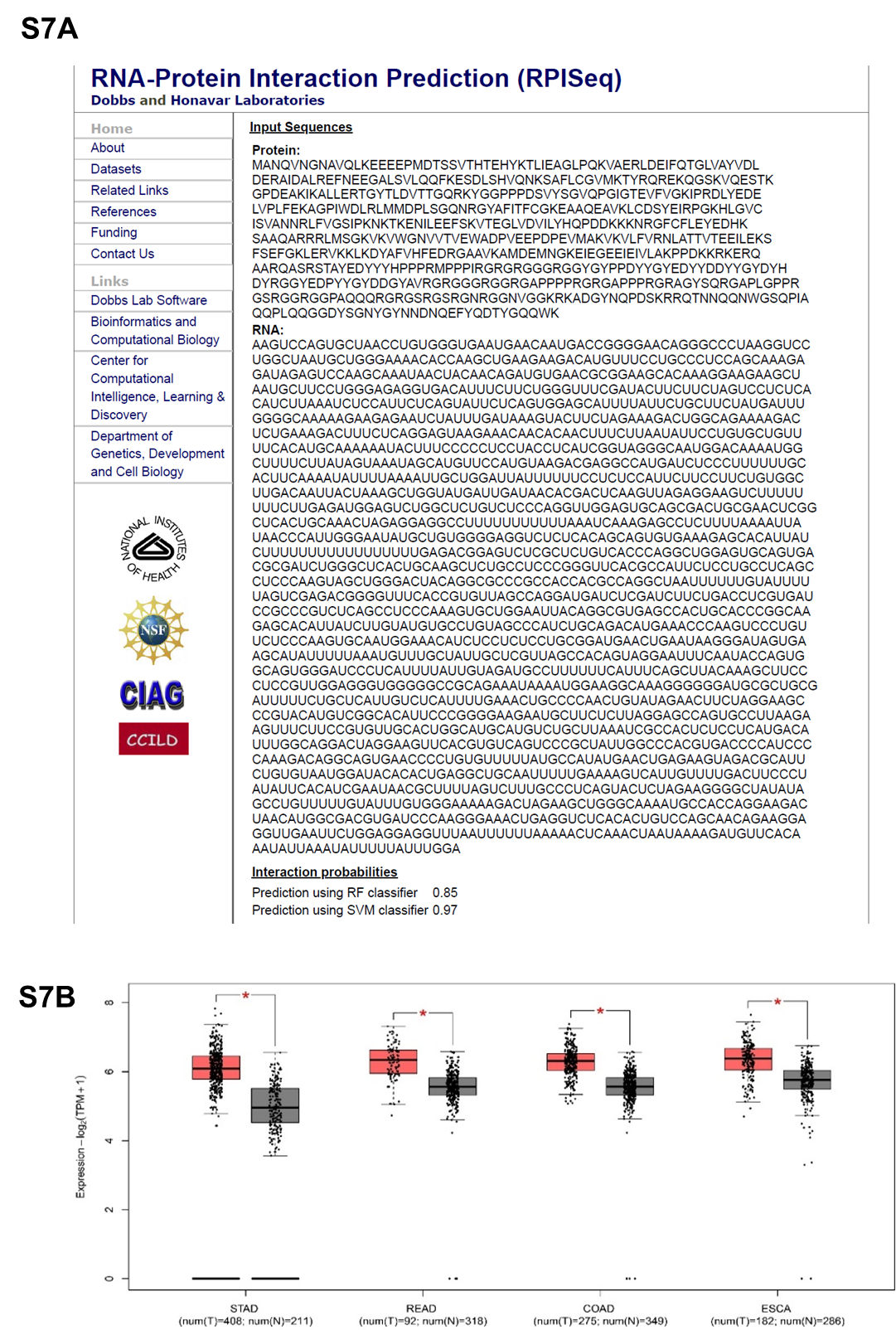


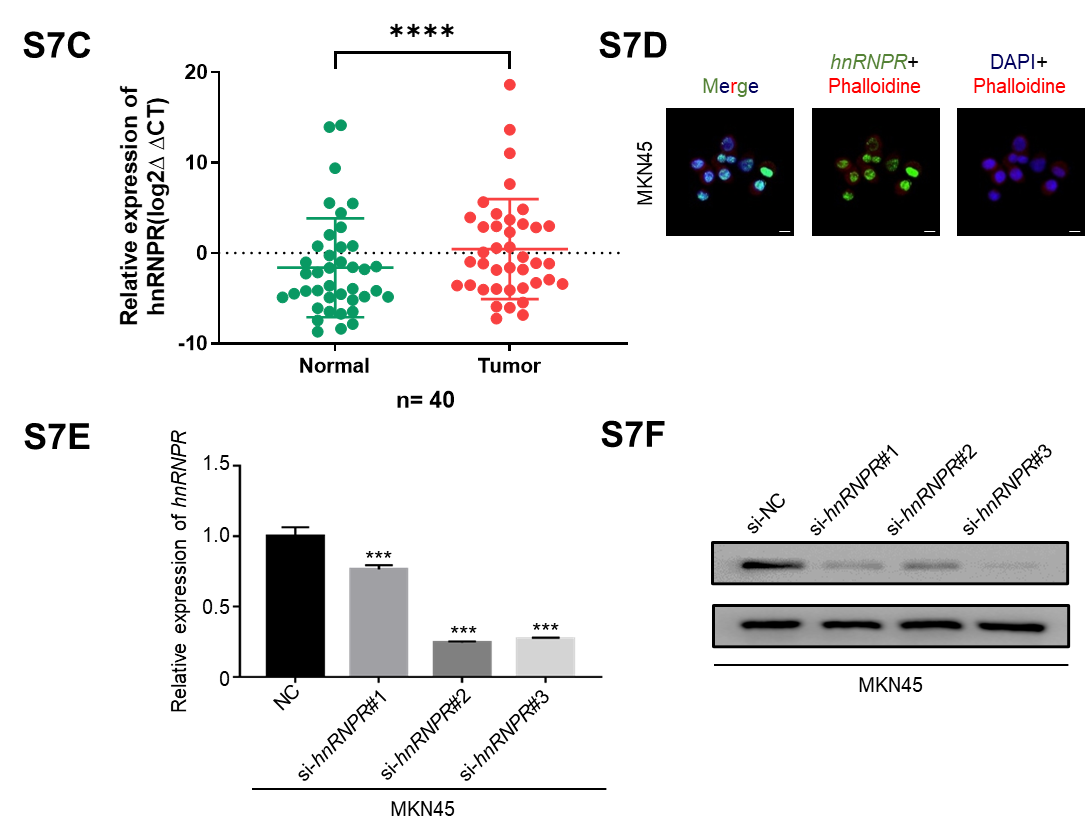


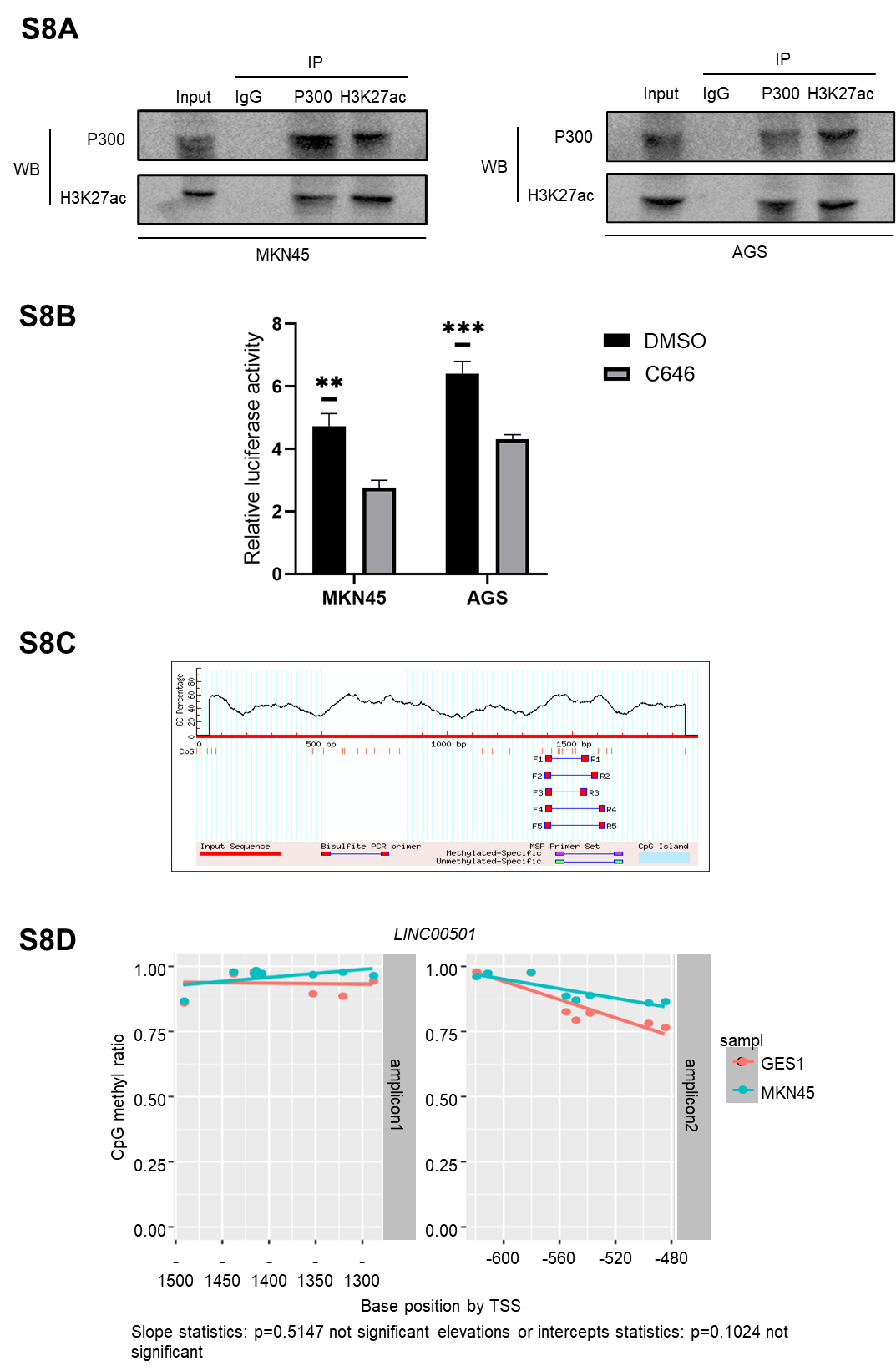


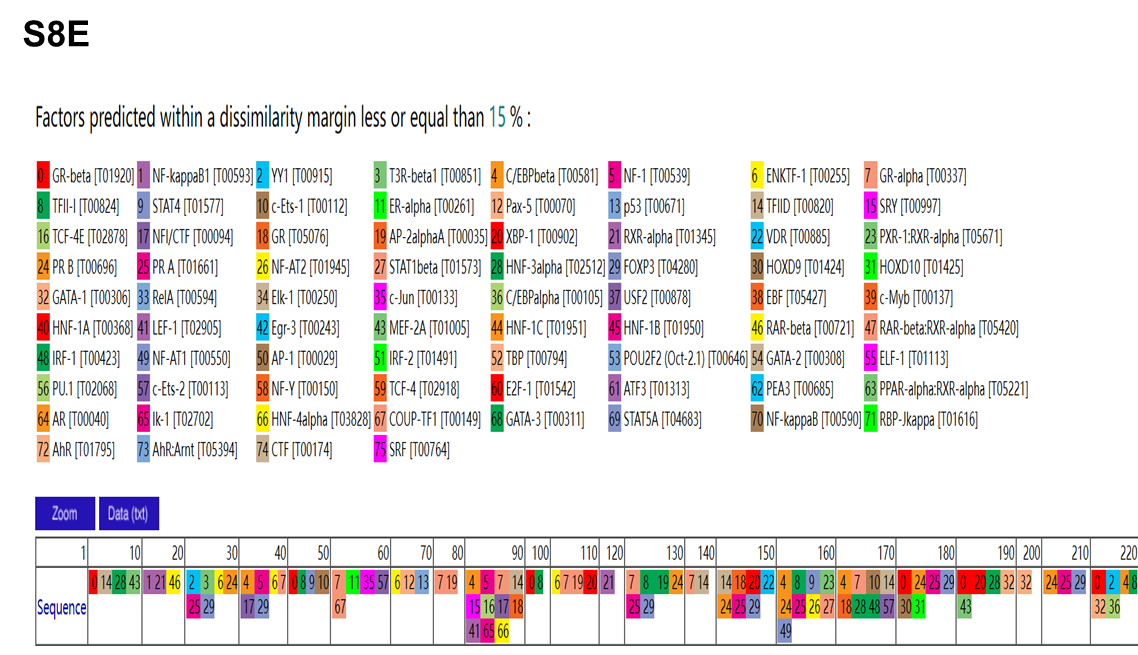


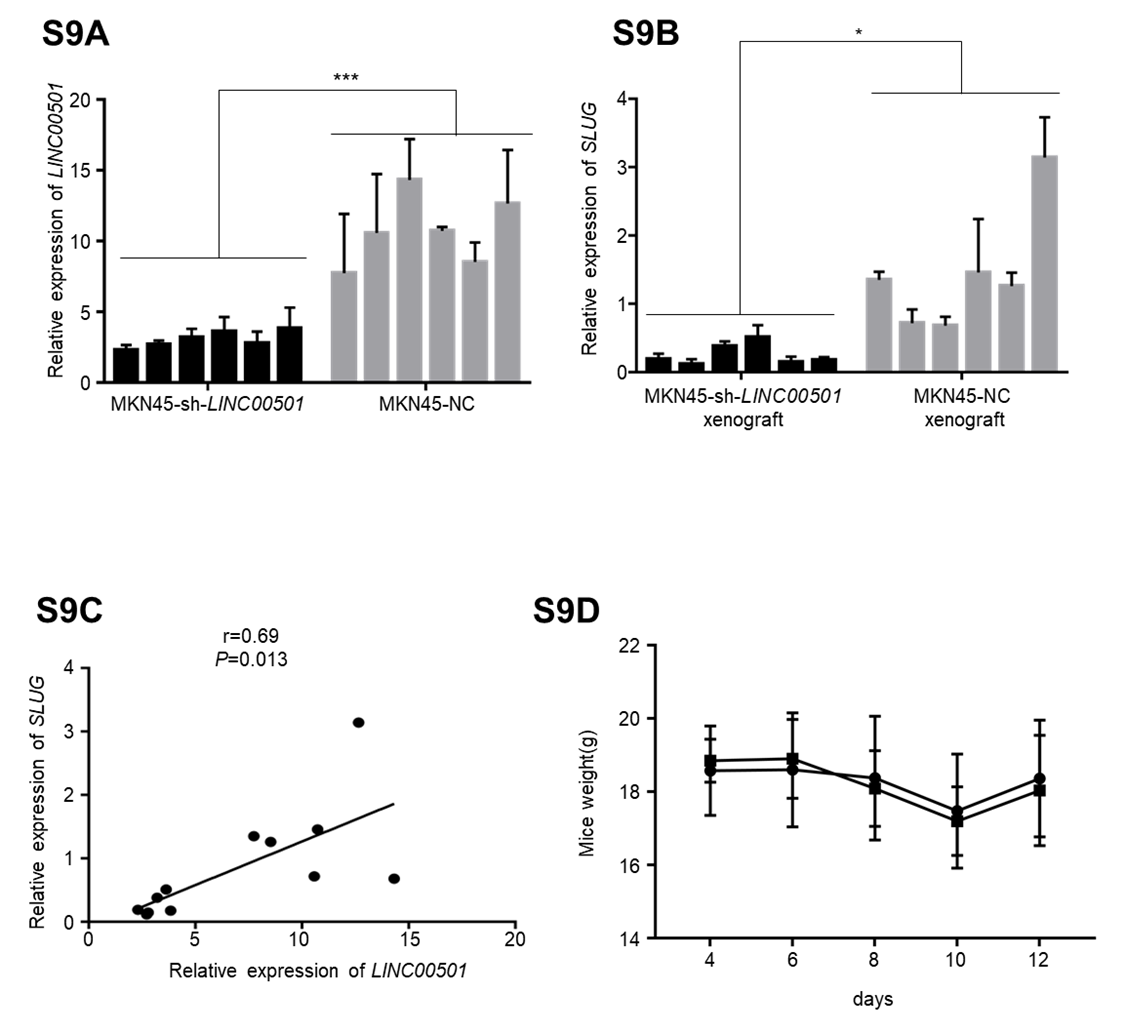


**Supplementary Table 1 The sequences of the primers for qRT-PCR**

| **Gene** | **Forward** | **Reverse** |
| --- | --- | --- |
| *LINC00501* | CCCTGTTCTCCCAAGTGCAA | TCCAACGGAGGGAAGCTTTG |
| *SH3PXD2A-AS1* | CAGTGAAGCCCACGAACGTA | GTTCACGATGTGAGCCCAGT |
| *FEZF1-AS1* | GTCACCTCGGTTCAGGTAGC | GCCGGCGTCTTCTAAATGGT |
| *DUXAP8* | GAGAAGCAGTGGTGGGTTCC | GAGCAACACAGATGAACCGC |
| *DUXAP10* | GGTGGGTTCCATGTGATGGT | GTCCAGTGCCTGTGAGGATT |
| *E-CADHERIN* | CACTGGGCTGGACCGAGAGAGTT | ACGCTGGGGTATTGGGGGCA |
| *VIMENTIN* | TTCCAAACTTTTCCTCCCTGAACC | TCAAGGTCATCGTGATGCTGAG |
| *N-CADHERIN* | TGCGGTACAGTGTAACTGGG | GAAACCGGGCTATCTGCTCG |
| *18S* | CGGACAGGATTGACAGATTGATAGC | TGCCAGAGTCTCGTTCGTTATCG |
| *β-ACTIN* | GTCATTCCAAATATGAGATGCGT | GCTATCACCTCCCCTGTGTG |
| *ZEB1* | CAGCTTGATACCTGTGAATGGG | TATCTGTGGTCGTGTGGGACT |
| *ZEB2* | GGAGACGAGTCCAGCTAGTGT | CCACTCCACCCTCCCTTATTTC |
| *SNAI1* | ACTGCAACAAGGAATACCTCAG | GCACTGGTACTTCTTGACATCTG |
| *SLUG* | TGTGACAAGGAATATGTGAGCC | TGAGCCCTCAGATTTGACCTG |
| *TWIST1* | CCGTGGACAGTGATTCCCAG | CCTTTCAGTGGCTGATTGGC |
| *U6* | CTCGCTTCGGCAGCACA | AACGCTTCACGAATTTGCGT |
| *TBL1XR* | CACCCGCTGCATTGATTTCTA | TACGGCATCTATCAGGGACAG |
| *KCNMB2* | GGTCCACAAAGGCTTTAGGC | ATGGCAAAAAGACCTCCGGTA |
| *ZMAT3* | CCTTACTTCAATCCCCGCTCT | CTTCGCCAGCTCCAACATTAC |
| *PIK3CA* | CCACGACCATCATCAGGTGAA | CCTCACGGAGGCATTCTAAAGT |
| *KCNMB3* | AGCAGGAACTGAGAGCAAGGG | AATGAGTCCAATGCCTCTCCC |
| *SLUG promoter primer 1* | GGCCAGCCTCTGGTGTTAAT | CCGCTTCCCCCTTCCTTTTT |
| *SLUG promoter primer 2* | GAACCCAGTCTAGTAACTGCAA | GTCTGCAATGGACAGAGATGC |
| *SLUG promoter primer 3* | TGTTCGGATGTAGGCACCTG | GCCATGGCGATATGTGTTTTCT |
| *SLUG promoter primer 4* | CATCAGCAGGTATCCGAGGG | GGGCATACGTGTTACTCGCT |
| *GAPDH promoter primer* | TGCTGAGTCACCTTCGAACC | ACTGTCTTCTCCCCGCAAAG |
| *hnRNPR* | CAACCTGGAATTGGAACGGAG | AGACGTAGATCCCAAATGGGTC |
| *CHIP primer for H3K27Ac/P300* | TTGCCAGGAGCATGTGAGTT | TAGCCTAGGGATGACGAGCA |
|  |  |  |

**Supplementary Table 2 The sequences of the siRNAs/shRNAs**

| **Gene** | **Sense** | **Antisense** |
| --- | --- | --- |
| *LINC00501* sh#1 | GCAAAGAGATAGAGTCCAAGC | - |
| *LINC00501* sh#2 | GCACCCGGCAAGAGCACATTA | - |
| *LINC00501* sh#3 | GCCACAGTAGGAATTTCAATA | - |
| *SLUG* si#1 | CCGGAUACUCCUCAUCUUUTT | AAAGAUGAGGAGUAUCCGGTT |
| *SLUG* si#2 | GCUUCAAGGACACAUUAGATT | UCUAAUGUGUCCUUGAAGCTT |
| *SLUG* si#3 | CCUGCACAAACAUGAGGAATT | UUCCUCAUGUUUGUGCAGGTT |
| *ZMAT3* si#1 | CCAGGUCUACAGGAACCUUTT | AAGGUUCCUGUAGACCUGGTT |
| *ZMAT3* si#2 | GCCCUGGAGGAGCUAUGUATT | UACAUAGCUCCUCCAGGGCTT |
| *ZMAT3* si#3 | GCAAGCCCAGGCUCAUUAUTT | AUAAUGAGCCUGGGCUUGCTT |
| *hnRNPR* si#1 | GCAGAAAGACUUGAUGAAATT | UUUCAUCAAGUCUUUCUGCTT |
| *hnRNPR* si#2 | GGACGUUAUUCUCUAUCAUTT | AUGAUAGAGAAUAACGUCCTT |
| *hnRNPR* si#3 | GCUACCCUCCAGAUUACUATT | UAGUAAUCUGGAGGGUAGCTT |

**Supplementary Table 3 Patient characteristics for microarray analysis.**

| **Patient** | **Gender** | **Age** | **TNM stage** | **Pathological grade** |
| --- | --- | --- | --- | --- |
| GC1/NAT1 | Male | 67 | T3N2M0 | G3 |
| GC2/NAT2 | Male | 68 | T4N2M0 | G2 |
| GC3/NAT3 | Male | 56 | T3N0M0 | G3 |
| GC4/NAT4 | Male | 60 | T4N3M0 | G2 |
| GC5/NAT5 | Male | 70 | T3N0M0 | G3 |
| GC6/NAT6 | Female | 60 | T4N0M0 | G3 |

**Supplementary Table 4 Correlation between LINC00501 and clinicopathological characteristics of gastric cancer patients in cohort1 (n=40).**

| **Characteristics** | **N (%)** | **LINC00501 expression** | |  |
| --- | --- | --- | --- | --- |
|  |  | **High** | **Low** | ***p*-value** |
| Gender |  |  |  | 0.735 |
| Female | 13 (32.5) | 7 | 6 |  |
| Male | 27 (67.5) | 13 | 14 |  |
| Age |  |  |  | 0.515 |
| >60 | 25 (62.5) | 11 | 14 |  |
| ≤60 | 15 (37.5) | 9 | 6 |  |
| AJCC stage |  |  |  | 0.018 |
| Ⅰ-Ⅱ | 14 (35) | 3 | 11 |  |
| Ⅲ-Ⅳ | 26 (65) | 17 | 9 |  |
| Metastasis |  |  |  | 0.077 |
| M0 | 29 (72.5) | 12 | 17 |  |
| M1 | 11 (27.5) | 8 | 3 |  |
| Lymphovascular  Invasion |  |  |  | 0.023 |
| Negative | 16 (40.0) | 4 | 12 |  |
| Positive | 24 (60.0) | 16 | 8 |  |

| **Characteristics** | **N (%)** | **LINC00501 expression** | |  |
| --- | --- | --- | --- | --- |
|  |  | **High** | **Low** | ***p*-value** |
| Gender |  |  |  | 0.92 |
| Female | 91 (29.9) | 51 | 40 |  |
| Male | 213 (70.1) | 118 | 95 |  |
| Age |  |  |  | 0.0502 |
| >60 | 145 (47.7) | 72 | 73 |  |
| ≤60 | 159 (52.3) | 97 | 62 |  |
| AJCC stage |  |  |  | <0.001 |
| Ⅰ-Ⅱ | 119 (39.1) | 41 | 78 |  |
| Ⅲ-Ⅳ | 185 (60.9) | 128 | 57 |  |
| Metastasis |  |  |  | 0.04 |
| M0 | 291 (95.7) | 158 | 133 |  |
| M1 | 13 (4.3) | 11 | 2 |  |

**Supplementary Table 5 Correlation between LINC00501 and clinicopathological characteristics of gastric cancer patients in cohort2 (n=304).**

**Materials and Methods**

**Microarray analysis**

6 pairs of gastric cancer/matched adjacent normal tissues were used for microarray analysis. Arraystar Human LncRNA Microarray V4.0 is designed for the global profiling of human LncRNAs and protein-coding transcripts, which is updated from the previous Microarray V3.0.Sample labeling and array hybridization were performed according to the Agilent One-Color Microarray-Based Gene Expression Analysis protocol (Agilent Technology). After data normalization of the raw data, the differentially expressed lncRNAs in gastric cancer with statistical significance were identified. The array data were available via GSE193109.

**Targeted bisulfite sequencing(TBS) PCR**

Gene-speciﬁc DNA methylation was assessed by a next-generation sequencing-based BSP, according to previously published method. ^[1]^ In brief, BSP primers were designed using the online MethPrimer software and the sequences are: Upstream: CACGACGCTCTTCCGATCT; Downstream: ACGTGTGCTCTTCCGATCT. 1 μg of genomic DNA was converted using the ZYMO EZ DNA Methylation-Gold Kit (Zymo Research, Irvine, CA, USA) and one twentieth of the elution products were used as templates for PCR ampliﬁcation with 35 cycles using KAPA HiFi HotStart Uracil+ ReadyMix PCR Kit (Kapa Biosystems, Wilmington, MA, USA). For each sample, BSP products of multiple genes were pooled equally, 5'-phosphorylated, 3'-dA-tailed and ligated to barcoded adapter using T4 DNA ligase (NEB). Barcoded libraries from all samples were sequenced on the Illumina platform.

**RNA extraction, reverse transcription and qRT-PCR**

According to the protocol, RNA was extracted with Trizol reagent (Vazyme, China). The reverse transcription of RNA into cDNA uses RT Master kit (Vazyme, China). The obtained cDNA was used for quantitative real-time PCR reaction with SYBR-Green PCR Master Mix (Vazyme, China).

**Western blot**

Proteins were separated by SDS-PAGE gel and transferred to the polyvinylidene fluoride (PVDF) membrane (Millibo, USA). After blocking with 5% skim milk for 60 minutes, the membrane was incubated at 4°C with the primary antibodies overnight. Placed the washed membrane in the secondary antibody, and incubated at room temperature for 60 min. Bio-Rad ChemiDoc XRS was used to detect protein signals.

**In situ hybridization (ISH)/ Fluorescence in situ hybridization (FISH)**

The ISH kit was purchased from Boster Biological (CA, USA). Paraffin gastric cancer tissue sections were deparaffinized and rehydrated with gradient alcohol-water solution, and endogenous peroxidase was inactivated with 3% H_2_O_2_. An appropriate amount of pepsin (1 mL 3% citric acid solution and two drops of concentrated pepsin) was added to tissue specimens and digested at 37°C for 10 min. The digestion was then quickly terminated with distilled water. After termination, the pre-hybridization solution was used at 37 °C for 4 hours, and hybridization was conducted using a digoxin-labeled *LINC00501* probe (Boster Biological, CA, USA) overnight at 40 °C. Till The next day, slides were washed in various concentrations of SSC solution. Biotinylated rat anti-digoxigenin, streptavidin–biotin complex, and biotinylated peroxidase were then added dropwise. After incubation at 37 ℃ for 60 min, slides were washed with ISH specific PBS. DAB color-developing solution was applied to testify the intensity of *LINC00501*. A four-tier system was utilized to score the maximum intensity/percentage of positive cells of the ISH staining in each tissue sample slide: 0 = negative (0%), 1 = weak (1-33%), 2 = moderate (34-66%), and 3 = strong (67-100%), the sum of the percentage score and intensity score is the score of ISH.

**Immunohistochemistry (IHC)**

The immunohistochemistry was conducted as described. ^[2]^ A four-tier system was utilized to score the maximum intensity/percentage of positive cells of the IHC staining in each tissue sample slide: 0 = negative (0%), 1 = weak (1-33%), 2 = moderate (34-66%), and 3 = strong (67-100%), the sum of the percentage score and intensity score is the score of IHC.

**Immunofluorescence**

The immunofluorescence was performed as described before. All antibodies used are described in Method.

**RNA decay assay**

The RNA decay velocity was testified by using actinomycin D. Specifically, 5ug/ml actinomycin D was administrated into gastric tumor cells, the relative expression level of *LINC00501* was testified with qRT-PCR in different time points-0h, 2h, 4h and 8h.

**CHX chase assay**

The SLUG protein degradation status was testified with 10 µM cycloheximide (CHX) and total protein was collected at different time points and subjected to immunoblotting for SLUG. To block the protein degradation, 20 µM MG132 was applied for 6 hours and the protein level of SLUG was then detected with western blot.

**Cell line and cell culture**

The human normal gastric epithelial cell line GES1 and GC cell lines (KATOIII, HGC27, AGS and MKN45) were purchased from the Chinese Academy of Sciences in Shanghai. Cells were cultured in RPMI (Roswell Park Memorial Institute) 1640 medium (Gibco, USA) for KATOIII, HGC27, AGS and DMEM (Dulbecco’s modified essential medium) for GES1 and MKN45 supplemented with 10% fetal bovine serum (Fetal bovine serum, FBS) (Gibco, USA) at 37 °C in a humidified atmosphere with 5% CO2.

**Animal experiment**

The Animal Health and Ethics Committee of Zhongnan Hospital of Wuhan University approved all operations of animal experiments. Twenty-four 4-6 weeks BALB/c athymic nude mice were purchased from Hubei Research Center of Laboratory Animals (Wuhan, China). For the tumor growth assay, 2×10^7^ gastric cancer cells in 100μl were subcutaneously injected into the right flank of the nude mice. After 8 days, we began to measure the size of tumor and calculated the tumor volume. The tumor volume was calculated with the equation: volume =1/2 ×(width^2^ × length). After 30 days, the weights of tumors were analyzed. And the RNA and protein were extracted. For tumor metastasis assay, twelve 4-6 weeks BALB/c athymic nude mice were randomly selected and 5×10^6^ gastric cancer cells were intrasplenic injected. 20 days after injection, the PET-CT was conducted to evaluate the liver metastasis conditions. The paraffin-embedded livers were serial sectioned and after hematoxylin-eosin (HE) staining, observe through a microscope.

**Plasmid construct**

The genomic sequences of the promoter region of human SLUG gene (-2000bp-TSS) and the serial truncated sequences were amplified by PCR and cloned into the XhoI/NotI site of pPro-RB-*SLUG* luciferase reporter vector (RiboBio, Guangzhou, China). All genomic products were confirmed by sequencing.

**Transwell invasion and migration assay**

The transwell invasion assay were conducted as described before. ^[2]^

For the migration assay,

1) Implant equal certain number of cells into 6-wells plate that after 24 hours of growth, they should reach 80%-90% confluence as monolayer.

2) Using 1 ml pipette tip to scratch the monolayer across the center of the well. Then wash the wells gently for twice with PBS to remove all the debris and change the medium that contains no FBS to exclude the effect of cell proliferation. Taking pictures in 3 random field each well and marked as 0-hour time point distance.

3) Waiting for another 24 hours, taking pictures in 3 random field each well and marked as 24-hour time point distance. Calculate the ratio between 24-hour distance and 0-hour distance.

**Transfection of siRNA of the target genes**

*SLUG, hnRNPR, ZMAT3* siRNAs were obtained from GenePharma (Shanghai, China). The Lipofectamine 2000 was applied to facilitate transfection (Invitrogen, USA), the final concentration of transfection was 50 nM. For further RNA experiments, the RNA was extracted 24 or 48 hours after transfection. The protein was extracted 48 or 72 hours after transfection.

**Antibodies and reagents**

The following antibodies and reagents were used: anti-E-CADHERIN, Proteintech (60335-1-Ig); anti-VIMENTIN, Proteintech (60330-1-Ig); anti-N-CADHERIN, Proteintech (66219-1-Ig); anti-β-ACTIN, Proteintech (66009-1-Ig); anti-ZEB1, Proteintech (66279-1-Ig); anti-ZEB2, Proteintech (67514-1-Ig), anti-SNAI1, Bioswamp (PAB33921); anti-SLUG, CST (#9585), anti-TWIST1, Proteintech (25465-1-AP); anti-hnRNPR, GeneTex (GTX16526); anti-PCCA, Proteintech (21988-1-AP); anti-DDX41, Proteintech (27500-1-AP); anti-ATAD3B, Proteintech (16610-1-AP); anti-RPN2, Proteintech (10576-1-AP); anti-H3K27Ac, CST (#8173), anti-P300, Abcam (ab275378); P300 inhibitor C646, MCE (HY-13823).

**Chromatin immunoprecipitation (ChIP) assay**

The ChIP assay was conducted by using CST® Enzyme Chromatin IP (#9003, USA) according to the manufacturer’s protocol. Briefly, the amount of 1×10^8^ cell was cross-linked with 37% methanol. After the reversal of cross-links, the cells were lysed and treated with ultrasonic. The chromatin was immunoprecipitated with specific antibodies and extracted, after that, qRT-PCR was utilized to assess the precipitated DNA level, primers for were listed in Supplementary Table 1.

**RNA immunoprecipitation (RIP) assay**

RIP assay was performed by using the RIP kit (Millibo, Massachusetts, USA). The collected tumor cells (the amount of tumor cells in each IP group is circa 5×10^7^) were lysed, the lysed-supernatant was co-cultured with the specific antibody. After being washed for six times, the RNA was extracted and reverse transcribed, then qRT-PCR was conducted to evaluate the amount of bound RNA.

**Targeted bisulfite sequencing(TBS) PCR**

Gene-speciﬁc DNA methylation was assessed by a next-generation sequencing-based BSP, according to previously published method[23].

**Chromatin isolation by RNA purification (ChIRP) assay**

The ChIRP assay was performed with ChIRP kit (GZSC-biotechnology) according to the manufacturer’s instructions. Briefly, a total of 1×10^7^ cells was fixed with methanol at a concentration of 1%. After the termination of cross-link with glycine, the cells were lysed and treated with ultrasonic. The synthesized biotin-labeled *LINC00501* probe and LacZ probe were applied to co-immunoprecipitated with lysate. Finally, the DNA was extracted and qRT-PCR was applied to quantify the precipitated DNA described as before.

**Dual luciferase reporter assay**

In brief, gastric cancer cells were co-transfected with 5μg luciferase reporter plasmid which contains the full-length/ serial truncated promoter sequence of SLUG and 5μg *LINC00501* plasmid in each well of 6-well-plate. 48 hours after the transfection, the luciferase activity was measured with the Dual-Luciferase Reporter Assay System (Promega, USA).

**Statistics**

All experimental data provided were expressed by means with SD unless otherwise specified. All statistical analyses used GraphPad Prism software (version 7.0, GraphPad software, United States) and SPSS statistical software (version 22.0, IBM SPSS, United States). Two-tailed Student’s t-tests or chi-square were used to compare the data between groups, when more than two groups were compared, one-way analysis of variance was applied. Values of P <0.05 are considered determined statistically significant.

**Authors' contributions**

RD, SW, BX participated in the design of the study; CY, LH, CP collected the clinical samples of gastric cancer; RD, LH, JZ, YF performed the *in vitro* and *in vivo* experiments; CL, JS, GH and CW provided assistance to the experiments; PZ, KL collected the clinical data; QP, RD, CY conducted the analysis of the data; RD, SW, CP wrote and modified the manuscript.

**List of abbreviations**

AJCC: American Joint Committee on Cancer

ANT: Adjacent normal tissue

AUC: Area under the curve

CHIP: Chromatin immunoprecipitation

CHIRP: Chromatin isolation by RNA purification

CHX: Cycloheximide

DAPI: 4′,6-diamidino-2-phenylindole

DMEM: Dulbecco’s modified Eagle medium

EMT: Epithelial mesenchymal transition

FBS: Fetal bovine serum

FISH: Fluorescence in situ hybridization

GC: Gastric cancer

GSEA: Gene set enrichment analysis

H3K27ac: Acetyl-Histone H3 (Lys27)

HE: Hematoxylin-eosin

IHC: Immunohistochemistry

ISH: In situ hybridization

LncRNA: Long noncoding RNA

NC: Negative control

NGS: Next generation sequence

qRT-PCR: Quantitative reverse transcription PCR

RBP: RNA binding protein

RFS: Recurrence free survival

RIP: RNA immunoprecipitation

ROC: Receiver operating characteristic

RPMI: Roswell Park Memorial Institute

SD: Standard deviation

TBS: Targeted bisulfite sequencing

[1] F. Gao, J. Zhang, P. Jiang, D. Gong, J. W. Wang, Y. Xia, M. V. Ostergaard, J. Wang, P. T. Sangild, BMC Genomics 2014, 15, 716, https://doi.org/10.1186/1471-2164-15-716.

[2] R. Dou, K. Liu, C. Yang, J. Zheng, D. Shi, X. Lin, C. Wei, C. Zhang, Y. Fang, S. Huang, J. Song, S. Wang, B. Xiong, Clin Transl Med 2021, 11 (12), e595, https://doi.org/10.1002/ctm2.595.
